# Antimicrobial Resistance and Comparative Genome Analysis of High-Risk *Escherichia coli* Strains Isolated from Ventilator-Associated Pneumonia Cases in Egyptian ICUs

**DOI:** 10.3390/microorganisms14071438

**Published:** 2026-06-30

**Authors:** Shaymaa Yusuf, Mona H. Abdel-Rahim, Omnia El-Badawy, Safy Hadiya, Amany G. Thabit, Radwa Abdelwahab, Heba A. Hammad, Shabaan H. Ahmed, Mohamed Samir, Xiaoqiang Liu, Douglas F. Browning, Sherine A. Aly

**Affiliations:** 1 Microbiology and Immunology Department, Faculty of Veterinary Medicine, Assiut University, Assiut 71515, Egypt; shaymaayusuf@yahoo.com; 2 Medical Microbiology and Immunology Department, Faculty of Medicine, Assiut University, Assiut 71515, Egypt; mona.hussein@aun.edu.eg (M.H.A.-R.); omniaalbadawy@aun.edu.eg (O.E.-B.); amanythabit@yahoo.com (A.G.T.); radwa.wahab418@gmail.com (R.A.); hebaali@aun.edu.eg (H.A.H.); shabaan@gmail.com (S.H.A.); 3 Department of Basic Medical Sciences, Badr University in Assiut (BUA), Assiut 19592, Egypt; 4 Assiut International Center of Nanomedicine, Al-Rajhy Liver Hospital, Assiut University, Assiut 71515, Egypt; ph.s.h.1989@gmail.com; 5 Microbiology and Immunology Department, School of Biotechnology, Badr University in Assiut, Assiut 19592, Egypt; 6 Department of Zoonoses, Faculty of Veterinary Medicine, Zagazig University, Zagazig 44519, Egypt; ms5711g@gre.ac.uk; 7 School of Science, Faculty of Engineering and Science, University of Greenwich, Medway campus, Kent ME4 4TB, UK; 8 College of Veterinary Medicine, Northwest A&F University, Xianyang 712100, China; liuxiaoqiang142@163.com; 9 Biosciences, Aston University, Aston Triangle, Birmingham B4 7ET, UK; 10 Aston Institute for Membrane Excellence, Aston University, Birmingham B4 7ET, UK

**Keywords:** *Escherichia coli*, Egypt, ventilator-associated pneumonia (VAP), antibiotic resistance, virulence, plasmids, whole-genome sequencing

## Abstract

*Escherichia coli* is increasingly recognised as an important cause of ventilator-associated pneumonia (VAP), particularly in intensive care units (ICUs) with high antimicrobial selective pressure. Unlike classical respiratory pathogens, ICU-associated *E. coli* often originates from the patient’s intestinal microbiota and harbours a complex mobilome enriched with antimicrobial resistance determinants. In this study, a total of 200 nosocomial endotracheal aspirate samples were aseptically collected from patients admitted to the Respiratory ICU at Assiut University hospital. Antimicrobial susceptibility testing, serotyping and screening for various virulence and antimicrobial resistance genes (e.g., extended-spectrum β-lactamase (ESBL) and carbapenems genes) were carried out. In total, *E. coli* isolates were recovered from 54/200 (27%) endotracheal aspirates, with a high prevalence of multidrug resistance (MDR) observed (74.1%). Resistance to β-lactams was common with phenotypic evidence suggestive of ESBL production detected in 64.8% of isolates. Genome sequencing of three MDR *E. coli* isolates confirmed that they carried multiple antimicrobial resistance genes, which included ESBL genes (e.g., *bla*_CTX-M-15_ and *bla*_TEM-1B_). Each strain was also found to be high-risk extraintestinal pathogenic *E. coli* (ExPEC) clones, belonging to either sequence type ST131 or ST405. These findings support an endogenous infection model for VAP, whereby ICU selective pressure favours highly mobile, multidrug-resistant *E. coli* lineages adapted for extraintestinal survival. The high production of ESBLs and the prevalence of carbapenemase genes highlight the urgent need for molecular surveillance and antimicrobial stewardship strategies for the control of such high-priority pathogens in this part of the world.

## 1. Introduction

Healthcare-associated infections remain a major threat to patient safety, particularly in intensive care units (ICUs), where critically ill patients are highly susceptible to opportunistic pathogens [1]. The ICU environment supports the persistence of clinically important pathogens, particularly the ESKAPE group (*Enterococcus faecium*, *Staphylococcus aureus*, *Klebsiella pneumoniae*, *Acinetobacter baumannii*, *Pseudomonas aeruginosa*, and *Enterobacter* spp.), which are well known for their ability to accumulate and disseminate antimicrobial resistance. Within this complex microbial landscape, *Escherichia coli* occupies a unique position as it is not only a commensal organism but also a cause of serious nosocomial infections [2]. *E. coli*’s capacity to induce ventilator-associated pneumonia (VAP) and other ICU-acquired infections reflects remarkable adaptability, driven by a diverse repertoire of virulence determinants and antimicrobial resistance mechanisms [2,3]. The co-occurrence of these traits poses additional challenges for clinical management and infection control, emphasising the need for a detailed molecular understanding of ICU-adapted *E. coli* strains [3].

Due to its eclectic ability to acquire virulence determinants, *E. coli* frequently causes diseases at various sites within the human body. In many instances, infection results in diarrhoea, which accounts for considerable global morbidity and mortality, particularly amongst children. In addition to diarrhoeagenic *E. coli*, some strains have acquired virulence genes that allow them to cause extraintestinal infections, such as urinary tract infections (UTIs), respiratory tract infections and sepsis, and have been termed extraintestinal pathogenic *E. coli* (ExPEC) [4,5]. In this context, *E. coli* is increasingly recognised as an emerging opportunistic pathogen in VAP, particularly among critically ill, mechanically ventilated patients, although it remains less frequently reported than classical ICU pathogens [6]. When present in the respiratory tract, especially as multidrug-resistant lineages, it is associated with poor clinical outcomes and limited therapeutic options [7]. The presence of endotracheal tubes further facilitates bacterial adhesion and biofilm formation, promoting persistent colonisation, immune evasion, and reduced antimicrobial efficacy [6,8]. Altogether, these factors highlight the clinical relevance of MDR *E. coli* in VAP, which is associated with prolonged mechanical ventilation, a longer ICU stay, and increased mortality in critically ill patients [7].

*E. coli* has been traditionally classified into four major phylogenetic groups, e.g., A, B1, B2, and D, based on their genetic makeup. Commensal isolates are generally associated with phylogroups A and B1, whereas pathogenic strains are more often linked to groups B2 and D [9,10]. Further classification is achieved by dividing strains into specific sequence types (STs) using multilocus sequence typing (MLST) based on the carriage of specific alleles of marker genes [11]. In parallel, serotyping based on O (lipopolysaccharide) and H (flagellar) antigens remains an important tool for epidemiological surveillance and the identification of clinically relevant lineages [12]. Phylogenetic background and serotype are frequently interrelated, as certain serotypes are consistently associated with specific phylogroups and pathogenic potential [13,14]. Together, these classification systems provide complementary insights into the epidemiology and virulence characteristics of *E. coli*.

The pathogenicity of *E. coli* is largely mediated by a wide array of virulence determinants that enable colonisation, invasion, immune evasion, and host persistence. Adhesion-associated genes such as *fimH*, *papA*, *papG*, *papGI*, *papGII*, and *papGIII* enable bacterial attachment to host tissues [15], while *afa*/*draBC* and *sfa*/*focDE* further promote colonisation and invasion. Importantly, several of these adhesins, particularly type 1 fimbriae (FimH) and S-fimbrial adhesins, are key virulence factors that facilitate binding to host cell receptors and abiotic materials. This interaction enables the establishment of biofilms, facilitating bacterial colonisation and increasing the risk of VAP in intubated patients [16]. Toxins encoded by *hlyA* and *cnf1* disrupt host cell membranes and enhance invasiveness [17]. Immune evasion is supported by genes including *kpsMII*, *traT*, and *ibseA* [18], whereas iron acquisition and metabolic fitness rely on genes such as *iutA*, *fyuA*, and *malX* [19]. Altogether, these determinants contribute to the adaptation of *E. coli* to the hostile ICU environment and facilitate colonisation in the respiratory tract.

Concurrently, *E. coli* serves as a major reservoir of multidrug resistance (MDR) genes. In particular, this encompasses strains resistant to third-generation cephalosporin antibiotics (such as ceftriaxone), due to the carriage of extended-spectrum β-lactamases (ESBLs) genes like *bla*_CTX-M_, *bla*_TEM_, and *bla*_SHV_ [20,21]. Furthermore, the carriage of carbapenemase genes, like the *Klebsiella pneumoniae* carbapenemase (*bla*_KPC_), New Delhi metallo-β-lactamase (*bla*_NDM_) and oxacillin hydrolysing enzymes (*bla*_OXA-48_-like), confer resistance to carbapenem antibiotics (e.g., imipenem), further compromising the efficacy of β-lactam antibiotics, which are at the frontline of fighting severe infections. [20,21,22,23]. The convergence of virulence and resistance within the same strain not only intensifies disease severity but also promotes persistence of MDR *E. coli* in ICU patients [24].

Against this background, the present study aimed to characterise *E. coli* isolates recovered from endotracheal aspirates of mechanically ventilated ICU patients, focusing on: (i) the prevalence of various antimicrobial resistance genes (ARGs); (ii) the distribution of phylogenetic groups and serotypes; and (iii) the repertoire of virulence genes that support adaptation to the respiratory tract. Increasing rates of MDR and carbapenem-resistant *E. coli* in intensive care units have become a major healthcare concern in Egypt and other low- and middle-income countries, but genomic surveillance data remain limited. Therefore, in addition, we use whole-genome sequencing to understand more about three of these MDR strains, identifying the ARG, plasmids and virulence determinants they carry, and use comparative genomics to place them in the context of the strains previously isolated and sequenced in Egypt. Furthermore, few studies have thoroughly investigated the relationship between antimicrobial resistance, virulence determinants, phylogenetic background, and genomic characteristics among respiratory isolates from mechanically ventilated patients, particularly in Egypt, despite the growing prevalence of MDR *E. coli* in ICUs. Thus, the integration of phenotypic and genotypic analyses in this study provides insights into the mechanisms underlying the persistence and pathogenic potential of *E. coli* in the ICU, offering a framework regarding antimicrobial stewardship measures, infection prevention strategies, and empirical therapy in critically ill patients.

## 2. Materials and Methods

### 2.1. Isolation and Characterisation of E. coli Strains from Endotracheal Aspirates

A total of 200 nosocomial endotracheal aspirates were aseptically collected from VAP patients admitted to the Respiratory ICU at Assiut University Hospital in 2020. Patients were diagnosed according to the Infectious Diseases Society of America (IDSA), defined as patients under mechanical ventilation for more than 48 h with new or progressive radiographic infiltrate, plus at least two of three clinical features (fever greater than 38 °C, leucocytosis or leucopenia, purulent secretions, new or worsening cough, dyspnoea, tachypnoea or impaired gas exchange) [25]. Exclusion criteria included patients who were mechanically ventilated for less than 48 h and those who did not fulfil the criteria of VAP. Samples were transported on ice and processed immediately or held at 4 °C for no longer than 24 h. *E. coli* isolates were recovered and identified using standard bacteriological methods [26]. Antimicrobial susceptibility was determined using the Kirby–Bauer disc diffusion method on Mueller–Hinton agar using a panel of β-lactams, aminoglycosides, fluoroquinolones, tetracycline, chloramphenicol, trimethoprim–sulfamethoxazole, and imipenem. Zone diameters were interpreted according to CLSI guidelines (2025) [27]. Isolates were classified as nondrug-resistant (NDR), single-drug-resistant (SDR), dual-antibiotic-resistant, or MDR, being defined as possessing resistance to one agent in three or more antimicrobial categories.

### 2.2. Detection of ESBLs and Carbapenemases

Phenotypic screening for ESBL production was performed by disc diffusion using oxyimino-cephalosporins according to CLSI criteria [27]. Confirmation was carried out using the combined disc method with cefoperazone and cefoperazone/sulbactam; an increase of ≥5 mm in inhibition zone diameter was considered positive [28]. Molecular detection of *bla*_CTX-M-3_-like, *bla*_CTX-M-14_-like, *bla*_TEM_, and *bla*_SHV_ genes was performed using PCR with the primer sequences detailed in Supplementary Table S1. The *bla*_CTX-M-3_-like primers target members of the CTX-M group 1 (CTX-M-1/CTX-M-3 lineage), while *bla*_CTX-M-14_-like primers target members of the CTX-M group 9 (CTX-M-14 lineage). Selected amplicons were sequenced, and variants were identified using the NCBI database (https://blast.ncbi.nlm.nih.gov/Blast.cgi (accessed on 25 March 2026)). For PCR reactions, positive and negative controls were incorporated to support the validity of data (Supplementary Figure S1). Carbapenemase activity was screened phenotypically using the modified Hodge test and the imipenem–EDTA combined disc test [29,30]. Carbapenemase genes (*bla*_IMP_, *bla*_VIM_, *bla*_NDM_ and *bla*_KPC_) were screened by PCR using the primers in Supplementary Table S1. Positive and negative PCR controls were incorporated to confirm the validity of data (Supplementary Figure S2).

### 2.3. Serotyping of E. coli Isolates

Serotyping was performed using monoclonal antisera (Statens Serum Institute, Copenhagen, Denmark) following the manufacturer’s instructions [31]. Isolates positive with polyvalent antisera were further tested using individual antisera. Heat-treated bacterial supernatants were mixed with antisera in microtiter plates, and reactions were read after overnight incubation. Carpet formation was considered positive, while button formation indicated a negative reaction.

### 2.4. Phylogenetic Analysis and Virulence Gene Profiling

Phylogenetic grouping was determined using the Clermont method [32]. PCR was used to detect virulence-associated genes, including *fimH*, *papA*, *papG (I–III)*, *afa/draBC*, *sfa/focDE*, *hlyA*, *cnf1*, *iutA*, *fyuA*, *kpsMII*, *traT*, *ibeA*, and *malX* using primers in Supplementary Table S1. Positive and negative controls were incorporated to support the validity of the PCR data (Supplementary Figure S3).

### 2.5. Genome Sequencing

Three *E. coli* isolates were selected for further characterisation based on their phylogenetic background, AMR phenotype, and virulence profiles. Two isolates belonged to phylogroup B2 and serogroup O25, features commonly associated with extra-intestinal pathogenic *E. coli* (ExPEC). To provide representation of another clinically relevant phylogenetic background, a third isolate belonging to phylogroup D was included. This isolate also exhibited MDR and carried multiple virulence-associated genes. The sequencing of *E. coli* strains was carried out by Microbes NG (https://microbesng.com/ (accessed on 25 March 2026)) using Illumina sequencing as before [33]. Trimmomatic 0.30 was used to trim reads using a Q15 sliding window quality cutoff [34]. SPAdes version 3.7 was used for genome assembly [35] and Prokka 1.11 for genome annotation [36]. The sequencing data for this project have been placed in DDBJ/ENA/GenBank (BioProject: PRJNA1460381) under the accession numbers E43: JBXYYI000000000, E106: JBXYYH000000000 and E110: JBXYYG000000000.

### 2.6. Bioinformatic Analysis of Genome Sequences

MLST 2.0 was used to determine bacterial sequence types [37], SerotypeFinder 2.0 was used for bacterial serotyping [38], plasmids were identified using PlasmidFinder 2.1 [39], ResFinder version 4.7.2 was used to identify antibiotic resistance determinants [40] and VirulenceFinder 2.0 and PathogenFinder 2 were used for virulence gene analysis [41,42,43] using software at the Center for Genomic Epidemiology (CGE) (http://www.genomicepidemiology.org/ (accessed on 25 March 2026)). The EzClermont in silico Clermont phylotyper was also used to determine the phylotype of each strain (https://ezclermont.hutton.ac.uk/ (accessed on 25 March 2026)) [44].

Artemis was used to visualise draft genomes [45], and genome comparisons were performed using the Proksee Server (https://proksee.ca/about (accessed on 25 March 2026)) [46], the Artemis Comparison Tool (ACT) [47] and the Basic Local Alignment Search Tool (BLAST 2.17) at NCBI (https://blast.ncbi.nlm.nih.gov/Blast.cgi (accessed on 25 March 2026)). Proksee [46] and ACT [47] were used to draw depictions of plasmid and genome organisation.

For phylogenetic analysis, Egyptian *E. coli* draft genomes were obtained from the NCBI Pathogen Detection Browser (https://www.ncbi.nlm.nih.gov/pathogens/ (accessed 25 March 2025)) [48] and the Enterobase Database (https://enterobase.warwick.ac.uk/ (accessed 25 March 2025)) [49]. Single-nucleotide polymorphism (SNP) analysis and phylogenetic tree construction were achieved using the bioinformatics pipeline at Solu Genomics (https://www.solugenomics.com/ (accessed on 25 March 2026)) [50]. Genomes were aligned to the reference genome of *E. coli* K-12 strain MG1655 (NC_000913) with Snippy v4.6.0 (https://github.com/tseemann/snippy (accessed on 25 March 2026)). Phylogenetic trees were constructed using IQ-TREE v2.3.6 [51] and visualised using NCBI Tree Viewer (https://www.ncbi.nlm.nih.gov/tools/treeviewer/ (accessed on 25 March 2025)) and FigTree v1.4.4 (https://github.com/rambaut/figtree/releases (accessed 25 March 2026)). Tree construction was validated by the inclusion of suitable *E. coli* reference genomes.

## 3. Results

### 3.1. Identification of E. coli Isolates and Antimicrobial Susceptibility Patterns

Out of the 200 endotracheal aspirates collected from patients admitted to the Respiratory ICU at Assiut University Hospital in 2020, 54 *E. coli* isolates (27%) were identified. Antimicrobial susceptibility testing against 16 antibiotics representing eight antimicrobial classes revealed high resistance rates across multiple agents. Within the penicillin group, resistance was highest to amoxicillin (81.5%), followed by piperacillin (70.4%) and amoxicillin–clavulanic acid (59.2%). Resistance to cephalosporins ranged from 68.5% to 81.5%, with the highest resistance observed for cefazolin (81.5%) and the lowest for ceftriaxone (68.5%). Among aminoglycosides, resistance to gentamicin and amikacin was 35.2% and 24%, respectively. Resistance rates to other antimicrobials included trimethoprim–sulfamethoxazole (72.2%), tetracycline (63%), levofloxacin (61.1%), chloramphenicol (22.2%), and imipenem (1.9%). Based on resistance profiles, 40 isolates (74.1%) were classified as MDR, four isolates (7.4%) as dual-antibiotic-resistant, while 10 isolates (18.5%) as NDR.

### 3.2. Phenotypic and Genotypic Detection of ESBL-Producing E. coli Isolates

Initial phenotypic screening identified 44 of 54 isolates (81.5%) as potential ESBL producers, while 10 isolates (18.5%) were classified as non-ESBL producers. Confirmatory testing using the combined disc method verified ESBL production in 35 of the 44 screened isolates, whereas 9 isolates were confirmed as non-ESBL producers (Supplementary Table S2). For downstream molecular characterisation, only the 44 isolates identified as potential ESBL producers during initial screening were included. This approach was adopted to focus on isolates with a higher likelihood of harbouring ESBL-associated resistance determinants and to enable detailed investigation into their genetic and phenotypic characteristics.

PCR screening of all 44 *E. coli* isolates detected 42 ESBL gene targets in 29 isolates (Supplementary Table S2). The most prevalent ESBL genes were *bla*_CTX-M-3_-like and *bla*_TEM_, each identified in 17 isolates (38.6%), followed by *bla*_CTX-M-14_-like in six isolates (13.6%) and *bla*_SHV_ in two isolates (4.5%). Sequencing identified multiple ESBL variants, reflecting notable genetic diversity among the isolates (Supplementary Table S2). Sequence analysis of *bla*_CTX-M-3_-like amplicons revealed three distinct CTX-M group 1 variants: *bla*_CTX-M-15_ (carrying the D240G substitution) detected in nine isolates, *bla*_CTX-M-109_ (with Q56R, D240G, and D288K substitutions along with a deletion of Gly289 and Leu290) detected in one isolate, and *bla*_CTX-M-216_ (possessing D240G and G289S substitutions) detected in seven isolates. Sequence analysis of the *bla*_CTX-M-14_-like amplicons identified two CTX-M group 9 variants: *bla*_CTX-M-17_ (with the E288K substitution) and *bla*_CTX-M-27_ (carrying the D240G substitution) in three isolates each. BLAST analysis of the *bla*_TEM_ sequences identified the reference *bla*_TEM-1_ (detected in six isolates) along with four variant types. These included an unclassified *bla*_TEM_ variant detected in six isolates (possessing a W286V substitution and insertion of three amino acids (NLS)), *bla*_TEM-214_ (with the I13F substitution) in three isolates, and *bla*_TEM-169_ (with M69L and W165G substitutions) and *bla*_TEM-190_ (carrying M69L, W165G, and N276D substitutions) each detected in one isolate. Sequence analysis of the two detected *bla*_SHV_ genes revealed identical amino acid substitutions (i.e., L35Q, G237S, and E238K), classifying them as *bla*_SHV-12_. Importantly, phenotypic ESBL expression showed strong concordance with the presence of ESBL genes (Supplementary Table S2).

### 3.3. Detection of Carbapenemases in E. coli Isolates

Metallo-β-lactamase production, as detected by the imipenem–EDTA combined disc test, was identified in 8 of 44 isolates (18.1%). PCR screening revealed the presence of the *bla*_NDM-1_ gene in 16 isolates (36.4%) (Supplementary Table S2). None of the isolates were positive for *bla*_KPC_, *bla*_VIM_, or *bla*_IMP_ genes.

### 3.4. Phylogenetic Grouping, Virulence Gene Profile and Serotyping of E. coli Isolates

Phylogenetic analysis of the 44 *E. coli* isolates showed that phylogroup A was the most prevalent (38.6%), followed by phylogroup B2 (31.8%), phylogroup D (25%), and phylogroup B1 (4.5%) (Supplementary Table S3). Serotyping of the 44 *E. coli* isolates revealed substantial diversity, with serotype O86a being the most prevalent, identified in 11 isolates (25%). Twelve isolates (27.2%) were non-typeable, and serotypes O1 and O125 were each detected in four isolates (9.09%), whilst O25, O114, and O146 were detected in two isolates each (4.54%). The remaining serotypes (O18, O111, O152, O78, O55, O102 and O167) were each identified in one isolate (2.27%) (Supplementary Table S3).

Virulence gene profiling revealed *traT* (77.3%) and *fimH* (77.3%) as the most frequently detected genes. Moderate prevalence was observed for *malX* (40.9%) and *iutA* (36.4%). Adhesion-associated genes, including *papA* (15.9%), *papGIII* (13.6%), *sfa/focDE* (13.6%), and *afa/draBC* (11.4%), were less common. Toxin-associated genes (*cnf1*, *hlyA*, *ibeA*) were detected in 9.1–11.4% of isolates. Notably, *papGI*, *papGII*, and *kpsMTII* were not detected in any isolate (Supplementary Table S3).

### 3.5. Genome Characterisation of E. coli Isolates E43, E106 and E110

To understand more about the strains that we have isolated, the genomes of three MDR strains, E43, E106 and E110, were sequenced using short-read Illumina whole-genome sequencing (Table 1). As predicted, E43 and E110 were phylotype B2, whilst E106 was phylotype D, with further phylogenetic analysis indicating that E43 and E110 were sequence type ST131 and E106 was ST405 (Table 1; Supplementary Table S3). It is of note that both ST131 and ST405 are considered globally dispersed high-risk ExPEC clones owing to their strong association with MDR, their ability to accumulate and disseminate resistance plasmids, and frequent involvement in healthcare-associated infections. Both lineages have previously been detected in Egypt [52,53,54]. Predictably, each strain possessed multiple ARGs, with genes that would enable resistance to β-lactams (e.g., *bla*_CTX-M-15_, *bla*_OXA-1_, and *bla*_TEM-1B_), carbapenems (*bla*_NDM-1_), aminoglycosides (*aac*(3)-IId, *aac*(6′)-Ib-cr, *aadA5*, *aph*(3″)-Ib, *aph*(3′)-VI, *aph*(6)-Id), fluoroquinolones (*aac*(6′)-Ib-cr), macrolides (*mphA*), sulphonamide (*sul1* and *sul2*), trimethoprim (*dfrA17* and *dfrB4*,), tetracyclines (*tetA* and *tetB*) and chloramphenicol (*catB3*) antibiotics being detected (Table 1) [40,55,56]. Each strain also carried point mutation in *gyrA*, *parC* and *parE*, which are associated with resistance to various quinolone antibiotics [40,55,56] (Table 1). Thus, the identification of these resistance determinants compares well with the AMR phenotypes observed for these strains (Table 1).

Unsurprisingly, analysis of each draft genome using PathogenFinder 2 indicated that all three strains were likely human pathogens (Table 1) [41,42,43]. Each strain carried a number of well-characterised virulence determinants, which included capsule biosynthetic genes *(kpsE* and *kpsMII*), glutamate decarboxylases acid resistance genes (*gadA*/*gadB*) [58], serum resistance genes (e.g., the *iss* lipoprotein [59] and *traT* outer membrane protein [60]) and iron scavenging systems (e.g., yersiniabactin, *fyuA*, *ipr1* and *ipr2*; aerobactin, *iucABCDiutA*; and *sitAB* [61]) (Table 1). In addition, both ST131 isolates E43 and E110 carried the *papA* pilin gene, the *sat* serine protease autotransporter toxin and the *usp* uropathogenic-specific protein, with E43 also carrying *papC* and *papG* and E110 carrying *afaABC* [18]. The ST405 isolate E106 also carried *hylE* and *hlyF* haemolysin genes as well as the salmochelin siderophore receptor gene, *iroN* (Table 1). It is of note that many of the virulence genes carried by these isolates are associated with ExPEC strains [18,52,54].

### 3.6. Characterisation of Plasmids Carried by Egyptian E. coli Isolates E43, E106 and E110

**Figure 1:**
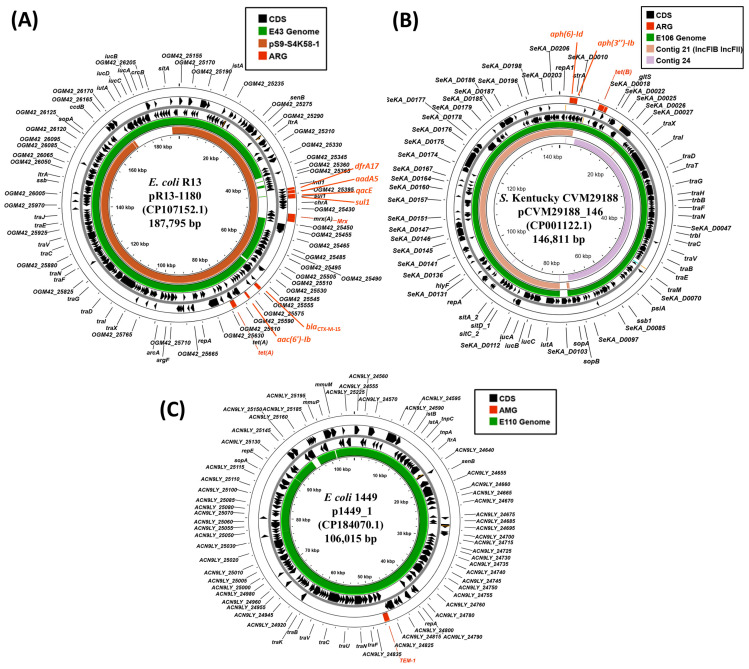
Analysis of the plasmids carried by *E. coli* isolates E43, E106 and E110. (**A**) The panel shows the comparison of *E. coli* strain R13 plasmid pR13-1180 (CP107152.1: human isolate) [62] with the draft genome of E43 and with plasmid pS9-S4K58-1 (human isolate: CP107123.1) [62] using ProkSee [46]. The genes (CDS) of pR13-1180 are displayed in the outer rings. The green and brown rings illustrate the BLAST results when the E43 draft genome and pS9-S4K58-1 are compared to pR13-1180. (**B**) The panel shows the comparison of plasmid pCVM29188_146 from *Salmonella enterica* serovar Kentucky CVM29188 (poultry: CP001122.1) [63] with the draft genome of E106 and E106 contigs 21 (79,866 bp) and 24 (65,526 bp) using ProkSee [46]. The genes (CDS) of pCVM29188_146 are displayed in the outer rings. The green, light brown and light purple rings depict the BLAST results when the sequences of the E106 draft genome and contigs 21 and 24 are compared with pCVM29188_146. (**C**) The panel shows the comparison of plasmid p1449_1 (CP184070.1: human isolate) from *E. coli* strain 1449 with the draft genome of E110 using ProkSee [46]. The outer two rings display the genes of p1149_1 (CDS), and the green ring illustrates the BLAST results when the E110 draft genome is compared to p1449_1. In all panels, the location of various ARGs is indicated.

Like most MDR *E. coli* strains, all three isolates carried multiple plasmid replicons, possibly suggesting that they carry a number of plasmids (Table 1). Due to the limitations of short-read sequencing, the complete sequence of plasmids could not be determined, as they were often encoded on multiple contigs. However, in spite of this, we were able to make predictions about the plasmids that each strain might harbour. For example, strain E43 carries five plasmid replicons (Table 1), and Blastn analysis indicated that E43 contigs 21 (Col156: 38,235 bp), 26 (IncFIA: 25,370 bp), 27 (IncFII: 24,910 bp) and 36 (IncFII: 10,220 bp) were identical (100% coverage/100% identity) to sections of plasmid pR13-1180 (CP107152.1: human isolate) [62] (Figure 1A; Supplementary Figure S4). Consistent with this, plasmid pR13-1180 carries Col156, IncFIA, IncFIB and two IncFII replicons and *aac*(6′)-Ib-cr, *bla*_CTX-M-15_, *bla*_OXA-1_, *catB3* and *tetA*, which are also both localised on E43 contig 27 (Figure 1A; Supplementary Figure S4). It is of note that strain R13, which carried pR13-1180, was isolated in Sweden in 2018 from a patient with a UTI and like E43 was sequence type ST131. Furthermore, a similar plasmid, pS9-S4K58-1 (CP107123.1: human isolate), was also isolated from *E. coli* strain S9 (also ST131) during that study (Figure 1A; Supplementary Figure S4) [62]. Thus, we propose that E43 might carry a similar multi-replicon plasmid to pR13-1180.

For strain E106, the IncFIB and IncFII replicons are both localised on contig 21 (79,866 bp), which also carries siderophore and iron transfer genes (*iucABCD-iutA* and *sitA*) and *aph*(3″)-Ib and *aph*(6)-Id aminoglycoside resistance genes (Table 1; Supplementary Figure S5). Blastn analysis indicated that contig 21 was very similar to *Salmonella enterica* serovar Kentucky CVM29188 plasmid pCVM29188_146 (poultry: CP001122.1) (97% coverage/99.98% identity) (Figure 1B; Supplementary Figure S5) [63]. Furthermore, E106 contig 24 (65,526 bp), which carries *tet* resistance and conjugational-transfer (*tra*) genes, was also similar to a large section of pCVM29188_146 (100% coverage/99.94% identity) (Figure 1B; Supplementary Figure S5). Interestingly, conjugational transfer of pCVM29188_146 was demonstrated to both *Salmonella* and *E. coli* strains, suggesting that this plasmid can be readily swapped between different members of the *Enterobacteriaceae* [63]. Thus, we propose that E106 might potentially carry a similar hybrid AMR-virulence plasmid.

Like E43, Egyptian isolate E110 carries five plasmid replicons (Table 1). Blastn analysis indicating that contigs 36 (IncFIA: 15,974 bp), 38 (IncFIB: 15,234 bp), and 41 (Col156: 10,625 bp) were identical to sections of plasmid p1449_1 (CP184070.1: human isolate) (100% coverage: 100% identity) (Figure 1C; Supplementary Figure S6). Plasmid p1449_1 was isolated from *E. coli* strain 1449 in the USA in 2016, and like E110 it was sequence type ST131. Thus, we propose that E110 could possibly carry a similar plasmid to p1449_1.

### 3.7. Phylogenetic Analysis of Egyptian E. coli Isolates

To understand more about the phylogeny of Egyptian *E. coli* strains, we searched the Enterobase Database [49] and the NCBI Pathogen Detection Browser [48] and identified 178 Egyptian *E. coli* with accessible draft genomes (Supplementary Table S4), increasing the number of strains we previously found [64]. Analysis of this data set indicated that the most abundant Egyptian sequence types were ST167, ST410, ST131, ST405, ST10 and ST361 (Table 2). Worryingly, the *bla*_NDM_ was the most prevalent carbapenemase derivative, and 53.5% (95/178) of sequenced Egyptian isolates were found to carry a carbapenemase gene, with 12 isolates carrying both *bla*_NDM_ and *bla*_OXA_ carbapenemases. Furthermore, for the two major sequence types identified (ST167 and ST410), carbapenemase gene carriage was remarkably high at 92.3% (36/39) and 82.6% (24/29), respectively (Table 2).

**Figure 2:**
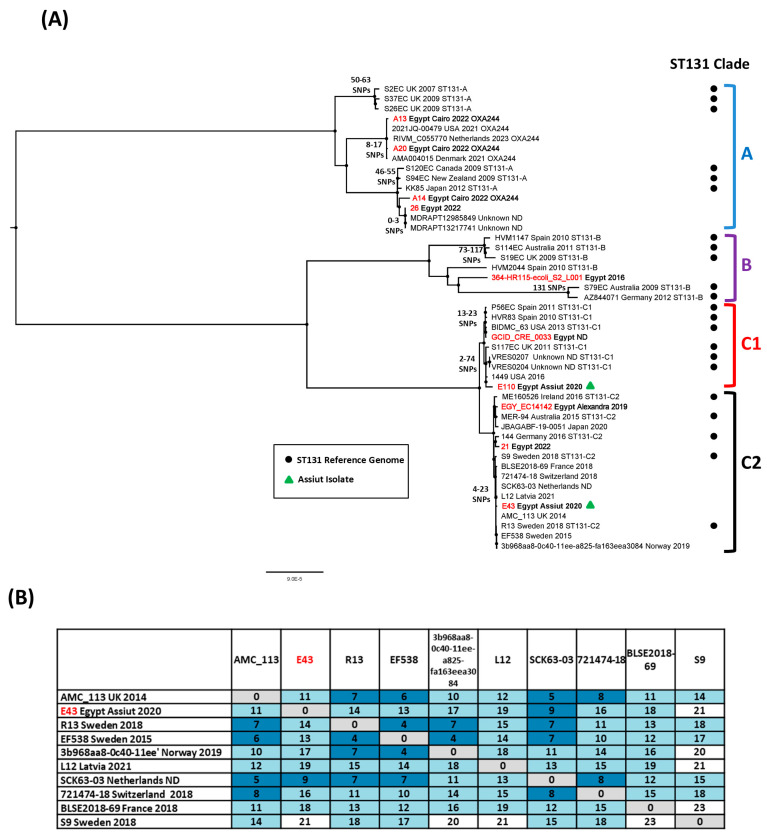
SNP analysis of Egyptian *E. coli* ST131. (**A**) The panel shows a phylogenetic analysis of ST131 *E. coli* strains. The ST131 strains isolated in this study are indicated by green triangles. SNP analysis of ST131 strains in Supplementary Table S5 was used to construct the tree. ST131 clades A, B, C1 and C2 are indicated, and ST131 reference genomes (indicated by black dots) have been used to confirm clade positioning [62,65,66]. Egyptian ST131 strains are highlighted in red and in bold, and at selected branch points the range of SNP differences is given. ND: no date. (**B**) SNP distance table showing the pairwise SNP differences for various strains from (**A**). Light blue shading denotes SNP differences between 20 and 10, whilst dark blue shading indicates SNP differences below 10.

Comparative genomics has classified *E. coli* sequence type ST131 into three major clades (A, B and C), which has been determined by the presence of various *fimH* alleles, serotypes, AMR point mutations and ARGs [62,65,66,67]. For example, clade A consists of strains with serotype O16:H5 carrying the *fimH41* allele, clade B contains serotype O25b:H4 with *fimH22*, and clade C contains serotype O25b:H4 with *fimH30*. Clade C has been further subdivided into major sub-clades C1 and C2, with C1 carrying point mutations associated with fluoroquinolone and C2 fluoroquinolone resistance coupled with carriage of *bla*_CTX-M-15_ [62,65,66,67]. In total, we identified ten Egyptian ST131 strains, including isolates E43 and E101 (Supplementary Tables S4 and S5). SNP analysis, coupled with phylogenetic analysis, indicated that Egyptian ST131 isolates were found in all three clades, clustering with previously identified ST131 reference genomes (Figure 2) [62,65,66]. Importantly, strain E43 was located in sub-clade C2, and SNP analysis indicated that it was very similar to the Swedish strains R13 (14 SNPs) and S9 (21 SNPs), which we previously identified [62] (Figure 1 and Figure 2). Furthermore, E43 was found to be close to other human isolates from the Netherlands (strain SCK63-03 (9 SNPs)), the UK (strain AMC_113 (11 SNPs), Sweden (EF538 (13 SNPs)), Switzerland (strain 721474-18 (16 SNPs)), Norway (strain 3b968aa8-0c40-11ee-a825-fa163eea3084 (17 SNPs)), France (BLSE2018-69 (18 SNPs) and Latvia (L12 (19 SNPs)) (Figure 2). As SNP threshold of ≤25 SNPs has previously been used to confirm outbreak strains within hospital settings [68,69], our data suggested that very similar ST131 strains were present within Egypt and throughout Europe.

**Figure 3:**
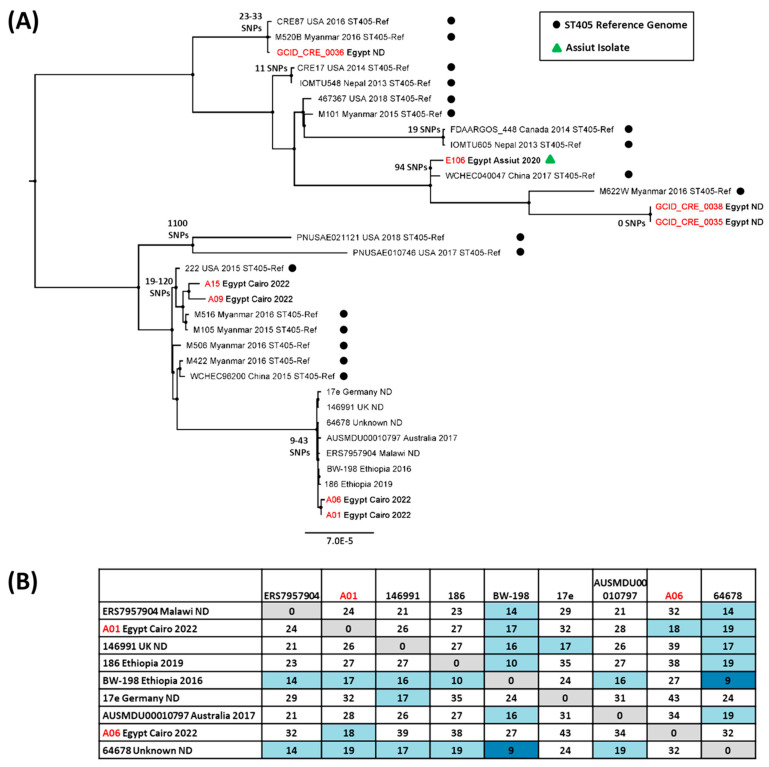
SNP analysis of Egyptian *E. coli* ST405 strains. (**A**) The panel shows a phylogenetic analysis of ST405 *E. coli* strains. The ST405 strain isolated in this study is highlighted by a green triangle. SNP analysis of ST405 strains in Supplementary Table S6 was used to construct the tree. ST405 reference genomes have been included to calibrate the tree, which are shown by black dots. Egyptian ST405 strains are highlighted in red and in bold. At selected branch points, the range of SNP differences is given. ND: no date. (**B**) SNP distance table showing the pairwise SNP differences for various strains from (**A**). Light blue shading denotes SNP differences between 20 and 10, whilst dark blue shading indicates SNP differences below 10.

With respect to sequence type ST405, we identified eight Egyptian isolates (Table 2; Supplementary Tables S4 and S6). Although *E. coli* ST405 has not been categorised into specific clades, we carried out SNP analysis coupled with phylogenetic analysis comparing Egyptian ST405 isolates with previously identified ST405 reference genomes (Figure 3) [70]. Interestingly, our results suggest that the ST405 may split into two separate clades with Egyptian ST405 isolates populating each clade. In particular, it is of note that Egyptian human isolates A01 and A06, which were isolated in Cairo in 2022 [53], were closely related, being separated by 18 SNPs, and formed a group of similar strains isolated from Ethiopia, Australia and Malawi, possibly suggesting strain circulation and that transmission events have occurred (Figure 3).

## 4. Discussion

In this study, *Escherichia coli* accounted for 27% (54/200) of isolates recovered from endotracheal aspirates of mechanically ventilated ICU patients, underscoring its substantial contribution to respiratory infections in this setting. Although *E. coli* has not historically been regarded as a primary pathogen in VAP, contemporary surveillance studies increasingly recognise the expanding role of Enterobacterales in ICU-acquired pneumonia, particularly in environments characterised by high antimicrobial pressure [71]. Large multicentre cohort analyses report *E. coli* as the causative pathogen in approximately 9–10% of VAP episodes, ranking behind *Pseudomonas aeruginosa* and *Klebsiella pneumoniae* yet remaining clinically significant [72]. In contrast, a prospective single-centre ICU study from Egypt documented a lower incidence of 5%, emphasising how institutional ecology, antibiotic consumption patterns, and infection-prevention practices may substantially influence pathogen distribution [73]. The markedly higher prevalence observed in our cohort likely reflects local factors such as prolonged mechanical ventilation, extensive empirical antibiotic use, and selective intestinal decontamination practices, all of which can promote intestinal overgrowth and selection of resistant Enterobacterales [74,75].

Phenotypic antimicrobial susceptibility testing revealed high resistance rates to β-lactams, ranging from 68.5% to 81.5%, with an overall multidrug resistance (MDR) rate of 74.1%. These findings align with contemporary ICU data, indicating MDR prevalence rates of 65–80% among Enterobacterales isolated from late-onset VAP [75,76]. Resistance to fluoroquinolones was also substantial, with 61.1% of isolates resistant by disk diffusion and 74.1% resistant by MIC testing. The discrepancy between disk diffusion and MIC results may reflect borderline susceptibility patterns and emphasises the importance of quantitative susceptibility assessment in ICU pathogens. High fluoroquinolone resistance among *Enterobacteriaceae* in critical care settings has been widely documented and is attributed to sustained selective pressure in patients exposed to repeated or prolonged antimicrobial regimens [77,78].

Despite low phenotypic resistance to carbapenems (1.9% for imipenem), molecular screening revealed *bla*_NDM-1_ in 36.4% of isolates. This apparent genotype–phenotype discordance is increasingly recognised in Enterobacterales, as carbapenemase genes may circulate in strains that do not yet exhibit high-level phenotypic resistance, particularly in the absence of porin loss or high expression levels [79]. Such silent carriage poses a significant epidemiological risk, as antibiotic pressure may rapidly select for phenotypic expression and clinical resistance. Extended-spectrum β-lactamase (ESBL) production was phenotypically confirmed in 64.8% of isolates, consistent with meta-analytic data reporting approximately 60% ESBL prevalence among clinical *E. coli* isolates in high-burden healthcare settings [80].

Molecular characterisation demonstrated predominance of *bla*_CTX-M_–type β-lactamases, with *bla*_CTX-M-15_ identified in 20.4% of isolates, making it the most prevalent ESBL variant in our cohort. This observation mirrors global surveillance findings that identify *bla*_CTX-M-15_ as one of the main ESBL genotypes in ICU-associated *E. coli*, largely due to its linkage with high-risk epidemic plasmids and successful clonal lineages [81,82]. The concurrent detection of multiple *bla*_CTX-M_, *bla*_TEM_ and *bla*_SHV_ variants within individual isolates reflects accumulation of resistance determinants under sustained antimicrobial pressure and ongoing microevolution [81,83,84]. The detection of *bla*_NDM-1_ in 36.4% of isolates places our ICU at the higher end of reported prevalence rates, consistent with global data and our analysis (Table 2) identifying *bla*_NDM_ carbapenemases as the most frequent carbapenemase gene in carbapenem-resistant *E. coli*, albeit with significant regional variability [85]. Phylogenetic clustering of *bla*_NDM_-positive isolates suggests local clonal dissemination, a pattern increasingly described in ICU molecular epidemiology studies [86].

Phylogenetic analysis revealed a heterogeneous population dominated by phylogroup A (38.6%), followed by B2 (31.8%), D (25%), and B1 (4.5%). Although community-associated ExPEC infections are frequently linked to phylogroup B2 [87], ICU-based studies increasingly report substantial phylogenetic heterogeneity, including significant representation of non-B2 lineages [88]. The predominance of phylogroup A in our cohort may reflect adaptation to the hospital environment and selection under antibiotic pressure. These findings support the widely accepted model that ExPEC strains often arise from endogenous intestinal reservoirs rather than exclusively from classical community-associated clones [87].

Genome sequencing of three of our VAP MDR strains identified that isolates E43 and E110 were phylotype B2 and ST131, whilst E106 was phylotype D and ST405 (Table 1; Supplementary Table S3). Consistent with their MDR phenotype, each strain possessed multiple ARGs (Table 1) including ESBLs (e.g., *bla*_CTX-M-15_ and *bla*_TEM-1B_) and E106 carried the *bla*_NDM-1_ carbapenemase gene. Furthermore, each strain carried well-characterised ExPEC virulence determinants, such as *pap* pilin genes, siderophore iron scavenging systems and toxins (Table 1), consistent with both ST131 and ST405 being global high-risk ExPEC clones [18,52,53,54]. The draft genomes of all three isolates also possessed multiple plasmid replicons (Table 1), though, due to the limitations of short-read sequencing, it was not possible to determine complete plasmid sequences. However, our analysis did suggest that each strain might carry a large multi-replicon AMR plasmid (Figure 1). For example, strain E43 potentially carries a plasmid similar to plasmid pR13-1180 (CP107152.1: human isolate) isolated from ST131 strain R13 in Sweden. Phylogenetic analysis was consistent with a close relationship between these strains (Figure 2), with E43 and R13 located in ST131 sub-clade C2, separated by only 14 SNP differences. Remarkably, E43 also clustered closely with strains from the Netherlands, UK, Sweden, Switzerland, Norway, France and Latvia (with a SNP difference range of 4 to 24) (Figure 2), and each strain appears to carry a plasmid analogous to pR13-1180 (Supplementary Figure S4C). This suggests that closely related ST131 strains have been present within Egypt and throughout Europe and that transmission events may have taken place. It is also of note that many of these strains were isolated from human urine or blood (Supplementary Table S5), highlighting the diverse niches that ExPEC ST131 can colonise and cause disease from. Indeed, the ST131 O25b:H4 lineage is a well-established gastrointestinal coloniser and a leading cause of extra-intestinal infections worldwide. Within this context, VAP-associated *E. coli* infections are typically endogenous, developing after airway or gastrointestinal colonisation with subsequent micro-aspiration of contaminated secretions, while biofilm formation on endotracheal tubes contributes to bacterial persistence. The *E. coli* ST131 lineage is further characterised by enhanced biofilm-forming capacity that may facilitate its persistence and success in healthcare-associated infections [8,89].

Whole-genome sequencing studies have demonstrated near-identical intestinal and pulmonary isolates, providing direct evidence of gut-to-lung translocation in VAP [90]. Accordingly, the detection of ExPEC-associated serogroups in our cohort likely reflects endogenous gut colonisation and subsequent aspiration under mechanical ventilation rather than true enteric pathogenesis. Overall, the integration of phylogenetic diversity, serotype heterogeneity, virulence profiles, and resistance determinants is consistent with an endogenous infection model for VAP. Furthermore, in critically ill, mechanically ventilated patients, intestinal barrier dysfunction, immune dysregulation, dysbiosis, and micro-aspiration facilitate migration of gut-derived bacteria into the lower respiratory tract [6,74,91]. These findings further support the gut as a major reservoir for ICU respiratory infections [90,92].

In conclusion, the *E. coli* isolates recovered from mechanically ventilated ICU patients represent a heterogeneous population of extraintestinal pathogenic strains adapted to respiratory infection [87,88]. These isolates are characterised by high MDR and ESBL prevalence, frequent plasmid-mediated resistance, and a virulence profile emphasising adhesion, serum resistance, and metabolic fitness rather than classical enteropathogenic or uropathogenic traits [81,83,93]. The presence of ExPEC-associated serogroups and virulence determinants is consistent with endogenous gut colonisation with subsequent aspiration under mechanical ventilation rather than enteric pathogenesis [90,94]. The combination of genotypic, phenotypic, and phylogenetic evidence supports the likelihood of an endogenous infection model for VAP [6,76,91]. These findings highlight the importance of *E. coli* as a clinically relevant, yet under-estimated etiologic agent of VAP, which may be overlooked in routine diagnostic and therapeutic algorithms. Importantly they also reinforce the critical need for antimicrobial stewardship and aspiration-focused infection-prevention strategies and suggest that surveillance of intestinal colonisation may help guide empirical therapy and limit the emergence of MDR ICU pathogens [74,95].

Several limitations should be acknowledged. Although the findings support an endogenous reservoir for VAP, the single-centre design may limit their generalizability to other ICU settings. Furthermore, the absence of paired gastrointestinal and respiratory isolates precluded direct assessment of gut-to-lung transmission, while the use of endotracheal aspirates cannot definitively differentiate true lower respiratory tract infection from contamination or device colonisation. As a result, alternative routes of acquisition, including device colonisation and healthcare-associated transmission, cannot be excluded. Despite these limitations, this study provides a comprehensive phenotypic and genotypic characterisation of ExPEC-associated VAP and offers valuable insight into the complex interplay between host factors, antimicrobial pressure, and bacterial adaptation that shapes the emergence of MDR respiratory pathogens in ICU settings.

## Figures and Tables

**Table 1:** Analysis of *E. coli* strains subjected to whole-genome sequencing in this study.

Strain	E43	E106	E110
Genome size	5,299,921 bp	5,554,700 bp	5,255,755 bp
Contigs	123	680	217
CDS ^a^	5018	5090	4951
GC%	50.73%	50.83%	50.76%
Phylotype ^b^	B2	D	B2
MLST ^c^	ST131	ST405	ST131
Serotype ^d^	O25:H4	O102:H6	O25:H4
*fimH* type ^e^	*fimH30*	*fimH27*	*fimH30*
Plasmid replicons ^f^	Col156, IncFIA, IncFIB, 2xIncFII	Col440I, IncFIB, IncFII	Col(MG828), Col156, IncFIA, IncFIB, IncFII
Resistance profile ^g^	MDR ^1,2,4,5,6^	MDR ^1,2,4,5,6,7,8^	MDR ^1,2,4,5,6,7^
Antibiotic resistance ^g^	AML, PI, CZ, CTR, CPD, CPZ, CIP	AML, PI, AM/C, CZ, CTR, CPD, CPZ, CIP, LEV, GEN, AK, TE, SXT, C	AML, PI, AM/C, CZ, CTR, CPD, CPZ, LEV, GEN, TE, SXT
Antimicrobial resistance genes ^h^	*aac*(6′)-Ib-cr, *bla*_CTX-M-15_, *bla*_OXA-1_, *catB3*, *tet(A)*	*aph*(3′)-VI, *aph*(3″)-Ib, *aph*(6)-Id, *bla*_NDM-1_, *bla*_CTX-M-15_, *bla*_TEM-1B_, *dfrB4*, *mph(A)*, *sul1*, *tet(A)*, *tet(B)*	*aac*(3)-IId, *aadA5*, *aph*(3″)-Ib, *aph*(6)-Id, *bla*_CTX-M-15_, *bla*_TEM-1B_, *dfrA17*, *mph(A)*, *sul1*, *sul2*, *tet(A)*
AMR point mutations ^h^	GyrA S83L, D87N ParC S80I, E84V, ParE I529L	GyrA S83L, D87N ParC S80I, ParE S458A	GyrA S83L, D87N, ParC S80I, E84V, ParE I529L
Virulence genes ^i^	*chuA*, *fdeC*, *fimH*, *fyuA*, *gad*, *hra*, *iha*, *irp1*, *irp2*, *iss iucC*, *iutA*, *kpsE*, *kpsMII_K5*, *malX*, *ompT*, *papA*, *papC*, *papG*, *sat*, *senB*, *shiB*, *sitAB*, *terC*, *traT*, *usp*	*air*, *chuA*, *eilA*, *fdeC*, *fimH*, *fyuA*, *gad*, *hlyE*, *hlyF*, *iroN*, *irp1*, *irp2*, *iss*, *iucC*, *iutA*, *kpsE*, *kpsMIII_K96*, *malX*, *ompT*, *shiA*, *sitAB*, *terC*, *traT*	*afaA*, *afaC*, *afaD*, *chuA*, *fdeC*, *fimH*, *fyuA*, *gad*, *iha*, *irp1*, *irp2*, *iss*, *iucC*, *iutA*, *kpsE*, *kpsMII*, *malX*, *ompT*, *papA*, *sat*, *senB*, *shiAB*, *sitA*, *terC*, *traT*, *usp*
Pathogen score ^j^	0.9757	0.9797	0.9789

^a^ CDSs: coding sequences. ^b^ Strain phylotype was determined using the EzClermont in silico Clermont phylotyper [44]. Software at CGE was used to identify: ^c^ the sequence type [37], ^d^ the serotype [38], ^e^ the *fimH* type [57], ^f^ the plasmid replicons [39], ^h^ various ARG and point mutations associated with AMR [40] and ^i^ the virulence determinants [41] each strain possessed. ^g^ Resistance profile. Antibiotics tested were: ^1^ penicillins (amoxicillin [AML], piperacillin [PI], amoxicillin/clavulanic acid [AM/C]); ^2^ cephalosporins (cefazolin [CZ], ceftriaxone [CTR], cefpodoxime [CPD], cefoperazone [CPZ]); ^3^ carbapenems (imipenem [IMP]); ^4^ fluoroquinolones (ciprofloxacin [CIP], levofloxacin [LEV]); ^5^ aminoglycosides (gentamicin [GEN], amikacin [AK]); ^6^ tetracyclines (tetracycline [TE]); ^7^ folate pathway inhibitors (trimethoprim/sulfamethoxazole [SXT]); ^8^ phenicol (chloramphenicol [C]). ^j^ PathogenFinder 2 was used to determine if strains were likely human pathogens [43], with scoring ranging from 0 to 1. Values that are closer to 1 indicate that the input organism was predicted as a human pathogen.

**Table 2:** Analysis of Egyptian *E. coli* strains by sequence type.

Sequence Type ^a^ (Phylotype ^b^)	Number of Strains per ST	Percentage of Strains per ST (n = 178)	Number of Strains Carrying *bla*_NDM_	Number of Strains Carrying *bla*_OXA_	Percentage of ST Carrying a Carbapenemase
**ST167** (A)	39	21.9%	34	3 (1) ^c^	92.3% (36/39) ^d^
**ST410** (C)	29	16.3%	21	13 (10) ^c^	82.6% (24/29) ^d^
**ST131** (B2)	10	5.6%	0	3	30% (3/10) ^d^
**ST405** (D)	8	4.5%	1	4	62.5% (5/8) ^d^
**ST10** (A)	7	3.9%	0	2	29.6% (2/7) ^d^
**ST361** (A)	6	3.3%	1	3	66.7% (4/6) ^d^
ST38, **ST155**, ST2165, ST8645, **ST--**	5	2.8%	1	3 (1) ^c^	nd
ST48	4	2.2%	0	0	0% (0/4) ^d^
**ST1715**, ST216, **ST224**, ST226, **ST648**, ST1011	3	1.7%	4	1	nd
**ST69**, ST156, ST617, **ST3541**, **ST6355**	2	1.1%	2	3	nd
ST43, **ST46**, ST58, ST450, ST457, **ST501**, ST515, ST744, ST997, ST11075, **ST1139**, ST1380, ST1421, ST1485, **ST1702**, **ST1722**, **ST3268**, ST4553, **ST4981**, ST7624, ST10825, **ST15578**	1	0.6%	2	6	nd
			**Total: 66**	**Total: 41 (12) ^c^**	

^a^ Sequence types presented in bold indicate that carbapenemase carriage was associated with that sequence type. ^b^ The phylotype of the major sequence types was determined using the EzClermont in silico Clermont phylotyper [44]. ^c^ Values in brackets indicate the number of strains carrying both *bla*_NDM_ and *bla*_OXA_ carbapenemase genes. ^d^ Values in brackets indicated the numbers of each sequence type carrying a carbapenemase gene. nd: not determined.

## Data Availability

This Whole Genome Shotgun project has been deposited at DDBJ/ENA/GenBank with the sequence data for *E. coli* strains (BioProject: PRJNA1460381) under the accession numbers E43: JBXYYI000000000, E106: JBXYYH000000000 and E110: JBXYYG000000000.

## Supplementary Materials

The following supporting information can be downloaded at: https://www.mdpi.com/article/10.3390/microorganisms14071438/s1, Figure S1: PCR analysis of ESBL genes carried by *E. coli* isolates; Figure S2: PCR analysis of carbapenemase genes carried by *E. coli* isolates; Figure S3: PCR analysis of *E. coli* virulence genes carried by *E. coli* isolates; Figure S4: Comparison of *E. coli* strain R13 plasmid pR13-1180 with the draft genome of *E. coli* E43; Figure S5: Analysis of contigs 21 and 24 from *E. coli* strain E106; Figure S6. Comparison of *E. coli* strain 1449 plasmid p1449_1 with the draft genome of *E. coli* E110; Table S1: PCR primers used in this study; Table S2: Phenotypic resistance profiles and genotypic characteristics of the 44 *E. coli* isolates identified as potential ESBL producers during initial screening (n = 44); Table S3: Phylogenetic, virulence and serotyping of 44 *E. coli* isolates; Table S4: The Egyptian *E. coli* draft genomes analysed in this study; Table S5: The *E. coli* ST131 genomes used for phylogenetic SNP analysis in this study; Table S6. The *E. coli* ST405 genomes used for phylogenetic SNP analysis in this study [33,37,40,46,47,48,49,50,52,53,54,62,63,64,65,66,70,96,97,98,99,100,101,102,103,104,105,106,107,108,109,110,111,112,113,114,115,116,117,118,119,120,121,122,123,124,125,126].
