# Supplementary material for: Antimicrobial Resistance and Comparative Genome Analysis of High-Risk Escherichia coli Strains Isolated from Ventilator-Associated Pneumonia Cases in Egyptian ICUs
Source: Microorganisms. 2026 Jun 30;14(7):1438. doi: 10.3390/microorganisms14071438 (PMC13414244; doi:10.3390/microorganisms14071438)

Edited by DFB on 25/6/2026

Antimicrobial resistance and comparative genome analysis of high-risk *Escherichia coli* strains isolated from ventilator-associated pneumonia cases in Egyptian ICUs.

Supplementary Material

Shaymaa Yusuf [1], Mona H. Abdel-Rahim [2], Omnia EL-Badawy[2,3], Safy Hadiya [4,5], Amany G. Thabit [2], Radwa Abdelwahab [2], Heba A. Hammad [2], Shabaan H. Ahmed [2], Mohamed Samir [6,7], Xiaoqiang Liu [8], Douglas F. Browning [9,10]* and Sherine A. Aly [2]*

[1] Microbiology and Immunology Department, Faculty of Veterinary Medicine, Assiut University, Assiut 71515, Egypt.
[2] Medical Microbiology and Immunology Department, Faculty of Medicine, Assiut University, Assiut 71515, Egypt.
[3] Department of Basic Medical Sciences, Badr University at Assiut (BUA), Assiut, Egypt.
[4] Assiut International Center of Nanomedicine, Al-Rajhy Liver Hospital, Assiut University, Assiut 71515, Egypt.
[5] Microbiology and Immunology Department, School of Biotechnology, Badr University in Assiut, Assiut, Egypt.
[6] Department of Zoonoses, Faculty of Veterinary Medicine, Zagazig University, Zagazig, Egypt.
[7] School of Science, Faculty of Engineering and Science, University of Greenwich, Medway campus, Kent, UK.
[8] College of Veterinary Medicine, Northwest A&F University, Yangling, China.
[9] Biosciences, Aston University, Aston Triangle, Birmingham B4 7ET, UK.
[10] Aston Institute for Membrane Excellence, Aston University, Birmingham, B4 7ET, UK.

Correspondence: d.browning@aston.ac.uk (D.F.B.); s-aly71@windowslive.com; s-aly71@aun.edu.eg (S.A.A.)

Supplementary Table S1. PCR primers used in this study.

Target gene	Primer sequence (all 5′ to 3′)	Ref.	Target gene	Primer sequence (all 5′ to 3′)	Ref.
bla$_{TEM}$	ATGAGTATTCAACATTTCCG<br>CTGACAGTTACCAATGCTTA	**[96]**	*papG*	CATTTATCGTCCTCAACTTAG<br>AAGAAGGGATTTTGTAGCGTC	**[97]**
bla$_{SHV}$	GGGTTATTCTTATTTGTCGC<br>TTAGCGTTGCCAGTGCTC	**[98]**	*papGI*	TCGTGCTGAGGTCCGGAATTT<br>TGGCATCCCCCAACATTATCG	**[99]**
bla$_{CTX-M-3}$ like	TCCCAGAATAAGGAATCCCAT<br>CCCATTCCGTTTCCGCTA	**[100]**	*papGII*	GGGATGAGCGGGCCTTTGAT<br>CGGGCCCCCAAGTAACTCG	**[101]**
bla$_{CTX-M-14}$ like	AAAAATGATTGAAAGGTGGTTGT<br>TTACAGCCCTTCGGCGATGA	**[102]**	*papGIII*	ACGCTGAATGCCACGTAAGA<br>TTTTGCATGGCTGGTTGTTC	**[103]**
**bla*$_{CTX-M-3}$-like	AATCACTGCGCCAGTTCACGCT<br>GAACGTTTCGTCTCCCAGCTGT	**[104]**	*sfaoc/DE*	CTCCGGAGAACTGGGTGCATCTTAC<br>CGGAGGAGTAATTACAAACCTGGCA	**[105]**
**bla*$_{CTX-M-14}$-like	TACCGCAGATAATACGCAGGTG<br>CAGCGTAGGTTCAGTGCGATCC	**[104]**	*afa/draBC*	GGCAGAGGGCCGGCAACAGGC/<br>CCCGTAACGCGCCAGCATCTC	**[99]**
**bla*$_{TEM}$	ATGAGTATTCAACATTTCCG<br>CTGACAGTTACCAATGCTTA	**[104]**	*iutA*	GGCTGGACATCATGGGAACTGG<br>CGTCGGGAACGGGTAGAATCG	**[106]**
**bla*$_{SHV}$	AACGGAACTGAATGAGGCGCT<br>TCCACCATCCACTGCAGCAGCT	**[107]**	*kpsMTII*	GCGCATTTGCTGATACTGTTG<br>CAATGATCGTATCGATGGGTTTT	**[103]**
bla$_{IMP}$	CTTGATGAAGGCGTTTATGTT<br>TAACCGCCTGCTCTAATGTAAG	**[108]**	*traT*	GGTGTGGTGCGATGAGCACAG<br>CACGGTTCAGCCATCCCTGAG	**[106]**
bla$_{VIM1}$	TCTACATGACCGCGTCTGTC<br>TGTGCTTTGACAACGTTCG	**[109]**	*cnf1*	AAGATGGAGTTTCCTATGCAGGAG<br>CATTCAGAGTCCTGCCCTCATTATT	**[110]**
bla$_{NDM-1}$	GGTTTGGCGATCTGGTTTTC<br>CGGAATGGCTCATCACGATC	**[111]**	*hlyA*	AACAAGGATAAGCACTGTTCTGGCT<br>ACCATATAAGCGGTCATTCCCGTCA	**[110]**
bla$_{KPC}$	ATGTCACTGTATCGCCGTCT<br>TTTTCAGAGCCTTACTGCCC	**[112]**	*ibeA*	TGAACGTTTCGGTTGTTTTG<br>TGTTCAAATCCTGGCTGGAA	**[113]**
fimH	TCGAGAACGGATAAGCCGTGG<br>GCAGTCACCTGCCCTCCGGTA	**[114]**	*malX*	GGACATCCTGTTACAGCGCGCA<br>TCGCCACCAATCACAGCCGAAC	**[115]**
papA	TGTTCAGTAATGAAAAAGAGGTTGT<br>TGAGCCGGAGGCTGAATTTT	**[103]**			

* Primers used for sequencing.

Supplementary Table S2. Phenotypic resistance profiles and genotypic characteristics of the 44 *E. coli* isolates identified as potential ESBL producers during initial screening (n = 44)

Strain	AMR profile [a]	Resistant Antibiotics [a]	ESBL[b]	CRE[c]	CTX-M group 1 (CTX-M-1/CTX-M-3 lineage)	CTX-M group 9 (CTX-M-14 lineage)	TEM	SHV	NDM
**E8**	MDR[1,2,3,4,7]	AML, PI, CZ, CTR, CPD, CPZ, IMP, CIP, LEV, SXT	+	-	CTX-M-15	CTX-M-17	-	-	NDM-1
**E9**	MDR[1,2,4,6,7]	AML, PI, CZ, CTR, CPD, CPZ, CIP, TE SXT	+	-		CTX-M-27	-	-	-
**E11**	MDR[1,2,4,7]	AML, PI, CZ, CTR, CPD, CPZ, CIP, SXT	+	-	-	-	-	-	-
**E12**	MDR[1,2,4,6,7,8]	AML, PI, AM/C, CZ, CTR, CPD, CPZ, CIP, LEV, TE, SXT, C	+	-	CTX-M-109	-	TEM*	-	-
**E13**	MDR[1,2,4,5,6,7]	AML, PI, CZ, CTR, CPD, CPZ, CIP, LEV, TE, SXT	+	-	CTX-M-216	-	TEM*	-	-
**E14**	MDR[2,6]	CZ, TE	-	-	-	-	-	-	-
**E15**	MDR[1,2,4,6,7]	AML, PI, AM/C, CZ, CTR, CPD, CPZ, CIP, LEV, TE, SXT	+	-	CTX-M-15	-	-	-	-
**E21**	MDR[1,2,4,5,67]	AML, PI, AM/C, CZ, CTR, CPD, CPZ, CIP, LEV, GEN, TE, SXT	+	-	CTX-M-216	-	-	-	-
**E26**	MDR[1,2,4,5,6,7]	AML, PI, AM/C, CZ, CTR, CPD, CPZ, CIP, LEV, GEN, TE, SXT	+	-	CTX-M-15	CTX-M-17	TEM-1	-	-
**E31**	MDR[1,2,4,5,6,7]	AML, PI, AM/C, CZ, CTR, CPD, CPZ, CIP, LEV, GEN, AK; TE, SXT	+	-	CTX-M-216	-	-	-	-
**E40**	MDR[1,2]	AML, CZ, CPD	-	-	-	-	-	-	-
**E41**	MDR[1,2,4,5,6,7]	AML, PI, AM/C, CZ, CTR, CPD, CPZ, CIP, LEV, GEN, TE, SXT	+	-	CTX-M-15	-	TEM-1	-	-
**E43**	MDR[1,2,4,5,6]	AML, PI, CZ, CTR, CPD, CPZ, CIP	+	-	CTX-M-15	-	-	-	NDM-1
**E47**	MDR[1,2,4,5,6,7]	AML, PI, AM/C, CZ, CTR, CPD, CPZ, CIP, LEV, TE, GEN, SXT	-	+	CTX-M-216	-	TEM*	-	-
**E49**	MDR[1,2]	AML, CZ	-	-	-	-	-	-	-
**E52**	MDR[1,2,4,6,7,8]	AML, PI, AM/C, CZ, CTR, CPD, CPZ, CIP, LEV, TE, SXT, C	+	-	CTX-M-216	CTX-M-17	-	-	-
**E58**	MDR[1,2,4,6,7]	AML, PI, CZ, CTR, CPD, CPZ; CIP, LEV, TE, SXT	+	-	-	-	-	-	-
**E61**	MDR[1,2,4,5,6,7,8]	AML, PI, CZ, CTR, CPD, CPZ, CIP, LEV, GEN, TE, SXT, C	+	-	CTX-M-15	-	TEM-1	-	-
**E74**	MDR[1,2,4,6,7,8]	AML, PI, AM/C, CZ, CTR, CPD, CPZ, CIP, LEV, TE, SXT, C	+	-		CTX-M-27	TEM-1	-	NDM-1
**E77**	MDR[1,2]	AML, CZ, CPD	-	-	-	-	-	-	-
**E83**	MDR[1,2,4,6]	AML, PI, AM/C, CZ, CIP, LEV, TE	-	-	CTX-M-216	-	-	-	-
**E84**	MDR[1,2,4,5,6,7,8]	AML, PI, AM/C, CZ, CTR, CPD, CPZ, CIP, LEV, GEN, AK, TE, SXT, C	+	+	CTX-M-15	-	-	-	-
**E85**	MDR[1,2,4,6,7,8]	AML, PI, AM/C, CZ, CTR, CPD, CPZ, CIP, LEV, TE, SXT, C	+	-	-	-	-	-	-
**E88**	MDR[1,2,4,5,7]	AML, PI, AM/C, CZ, CTR, CPD, CPZ, CIP, LEV, GEN, AK, SXT	+	-	CTX-M-15	-	TEM-169	-	-
**E90**	MDR[1,2,4,6,7]	AML, PI, AM/C, CZ, CTR, CPD, CPZ, CIP, LEV, TE, SXT	+	-	CTX-M-216	-	-	-	NDM-1
**E91**	MDR[1,2,4,5,7]	AML, PI, AM/C, CZ, CTR, CPD, CPZ, CIP, LEV, AK, SXT	+	-	-	-	-	-	-
**E92**	MDR[1,2,4,5,6,7,8]	AML, CZ, CPD, GEN, TE, LEV, SXT, C	-	-	-	-	TEM-1	SHV-12	-
**E95**	MDR[1,2,4,5,6,7]	AML, PI, AM/C, CZ, CTR, CPD, CPZ, CIP, LEV, GEN, TE, SXT	+	-		CTX-M-27	TEM-1	-	-
**E96**	MDR[1,2,4,6, 7]	AML, PI, AM/C, CZ, CTR, CPD, CPZ, CIP, LEV, TE, SXT	+	-	-	-	-	-	-
**E102**	MDR[1,2,4,5,6,7]	AML, PI, AM/C, CZ, CTR, CPD, CPZ, CIP, LEV, AK, TE, SXT	+	-	-	-	TEM-214	-	NDM-1
**E105**	MDR[1,2,4,5,6,7,8]	AML, PI, AM/C, CZ, CTR, CPD, CPZ, CIP, LEV, GEN, TE, SXT, C	+	-	-	-	-	SHV-12	NDM-1
**E106**	MDR[1,2,4,5,6,7,8]	AML, PI, AM/C, CZ, CTR, CPD, CPZ, CIP, LEV, GEN, AK, TE, SXT, C	+	+	-	-	TEM*	-	NDM-1
**E107**	MDR[1,2,4,5,6,7]	AML, PI, AM/C, CZ, CTR, CPD, CPZ, CIP, LEV, GEN, AK, TE, SXT	+	-	-	-	-	-	NDM-1

**E108**	MDR[1,2,4,5,6,7]	AML, PI, AM/C, CZ, CTR, CPD, CPZ; CIP, LEV, GEN, AK, TE, SXT	+	+	-	-	-	-	NDM-1
**E109**	MDR[1,2,4,6,7]	AML, PI, AM/C, CZ, CPD, CPZ, LEV, TE, SXT	-	+	-	-	-	-	NDM-1
**E110**	MDR[1,2,4,5,6,7]	AML, PI, AM/C, CZ, CTR, CPD, CPZ, LEV, GEN, TE, SXT	+	-	CTX-M-15	-	-	-	NDM-1
**E111**	MDR[1,2,4,6,7]	AML, PI, AM/C, CZ, CTR, CPD, CPZ; CIP, LEV, TE, SXT	+	-	-	-	-	-	NDM-1
**E112**	MDR[1,2,4,5,7,8]	AML, PI, AM/C, CZ, CTR, CPD, CPZ, CIP, LEV, GEN, AK, SXT, C	+	+	-	-	-	-	NDM-1
**E113**	MDR[1,2,4,7]	AML, PI, AM/C, CZ, CTR, CPD, CPZ; CIP, LEV; SXT	+	-	-	-	TEM*	-	-
**E118**	MDR[1,2,4,6,7]	AML, PI, AM/C, CZ, CTR, CPD, CPZ, CIP, LEV, TE, SXT	+	+	-	-	TEM-214	-	NDM-1
**E119**	MDR[1,2,6,7]	AML, PI, AM/C, CZ, CTR, CPD, CPZ, TE, SXT	-	-	-	-	-	-	-
**E122**	MDR[1,2,4,5,6,7,8]	AML, PI, AM/C, CZ, CTR, CPD, CPZ; CIP, LEV, GEN, AK, TE, SXT, C	+	-	-	-	TEM-214	-	-
**E123**	MDR[1,2,4,5,6,7]	AML, PI, AM/C, CZ, CTR, CPD, CPZ; CIP, LEV, GEN, AK, TE, SXT	+	-	-	-	TEM*	-	NDM-1
**E124**	MDR[1,2,3,4,5,6,7]	AML, PI, AM/C, CZ, CTR, CPD, CPZ, IMP, CIP, LEV, GEN, AK, TE, SXT	+	+	-	-	TEM-190	-	NDM-1
**Strain Totals**			**35/44** (79.5%)	**8/44** (18.1%)	**17/44** (38.6%)	**6/44** (13.6%)	**17/44** (38.6%)	**2/44** (4.5%)	**16/44** (36.4%)

[a]AMR Profile: [1] Penicillins (amoxicillin [AML], piperacillin [PI], amoxicillin/clavulanic acid [AM/C]); [2] Cephalosporins (cefazolin [CZ], ceftriaxone [CTR], cefpodoxime [CPD], cefoperazone [CPZ]); [3] Carbapenems (imipenem [IMP]); [4] Fluoroquinolones (ciprofloxacin [CIP], levofloxacin [LEV]); [5] Aminoglycosides (gentamicin [GEN], amikacin [AK]); [6] Tetracyclines (tetracycline [TE]); [7] Folate pathway inhibitors (trimethoprim/sulfamethoxazole [SXT]); [8] Phenicol (chloramphenicol [C]).
[b] ESBL: phenotypic ESBL detection was carried out using the combined disc diffusion assay.
[c] CRE: Metallo-β-lactamase production was detected using the imipenem–EDTA combined disc assay.

Supplementary Table S3. Phylogenetic, virulence and serotyping of 44 *E. coli* isolates.

Strain ID	Phylotype	O serotype	*fimH*	*papA*	*papG*	*papGI*	*papGII*	*papGIII*	*afa/draBC*	*sfa/focDE*	*hlyA*	*cnf1*	*iutA*	*fyuA*	*kpsMTII*	*traT*	*ibeA*	*malX*
E8	D	O86a	+	-	-	-	-	-	-	-	-	-	-	+	-	-	-	-
E9	B2	O1	+	+	-	-	-	-	-	-	-	-	+	+	-	+	-	+
E11	B2	O86a	+	-	-	-	-	+	-	-	+	-	+	+	-	+	-	+
E12	A	NA	-	-	-	-	-	-	-	+	+	-	+	-	-	+	-	+
E13	B2	NA	+	-	-	-	-	-	-	-	-	-	-	+	-	+	-	+
E14	D	O86a	+	-	-	-	-	-	-	-	-	+	-	-	-	-	-	-
E15	B2	O1	+	-	-	-	-	-	-	-	-	-	-	+	-	+	-	+
E21	A	NA	-	-	-	-	-	-	-	-	-	+	-	+	-	+	-	-
E26	B2	O1	+	+	-	-	-	-	+	-	-	-	-	+	-	+	-	+
E31	A	O18	-	-	-	-	-	-	-	-	-	-	-	-	-	+	-	-
E40	A	O86a	-	-	-	-	-	-	-	-	-	+	-	-	-	+	-	-
E41	A	NA	-	-	-	-	-	-	-	-	-	-	-	-	-	+	-	-
E43	B2	O25	+	-	+	-	-	+	-	-	-	-	+	+	-	+	+	+
E47	D	O86a	+	-	-	-	-	-	-	-	-	-	-	+	-	+	-	+
E49	B2	NA	+	+	-	-	-	-	-	+	-	+	+	+	-	-	-	+
E52	D	NA	+	-	-	-	-	-	-	-	-	-	-	+	-	+	-	+
E58	D	O125	+	-	-	-	-	+	-	-	-	-	+	+	-	+	-	+
E61	B1	O125	+	-	-	-	-	-	-	-	-	-	-	-	-	+	+	-
E74	A	NA	+	-	-	-	-	-	-	-	-	-	-	-	-	+	-	-
E77	D	O86a	+	-	-	-	-	-	-	-	+	-	-	+	-	+	-	-
E83	B2	O86a	+	-	-	-	-	-	-	-	-	-	-	-	-	+	-	-
E84	B2	O86a	+	+	-	-	-	+	+	-	-	-	+	+	-	+	-	+
E85	B2	O114	+	+	-	-	-	-	-	-	+	-	+	+	-	+	-	+
E88	A	NA	+	-	-	-	-	-	-	-	-	-	+	-	-	+	-	-
E90	D	O86a	+	-	-	-	-	+	-	+	-	+	-	+	-	-	-	-
E91	B2	O111	+	-	-	-	-	-	+	+	-	-	+	+	-	+	+	+
E92	A	O146	+	-	-	-	-	-	-	-	-	-	+	-	-	+	-	-
E95	A	NA	+	-	-	-	-	-	-	-	-	-	-	-	-	+	-	-
E96	A	O55	+	-	+	-	-	+	+	+	-	-	+	+	-	-	+	-
E102	B2	O114	-	-	-	-	-	-	-	-	-	-	-	-	-	-	-	-
E105	B1	O125	+	-	-	-	-	-	-	-	-	-	-	-	-	+	-	-
E106	D	O102	+	+	-	-	-	-	-	-	-	-	+	-	-	+	-	+
E107	A	O86a	+	-	-	-	-	-	-	-	-	-	-	-	-	-	-	-
E108	A	O152	-	-	-	-	-	-	-	-	-	-	-	-	-	+	-	-
E109	A	NA	-	-	-	-	-	-	-	-	-	-	+	+	-	+	-	+
E110	B2	O25	+	+	+	-	-	-	+	+	-	-	+	+	-	+	-	+
E111	B2	O78	+	-	-	-	-	-	-	-	-	-	-	+	-	+	-	-
E112	A	O125	+	-	-	-	-	-	-	-	-	-	-	-	-	-	-	-
E113	A	NA	-	-	-	-	-	-	-	-	-	-	-	-	-	+	-	-
E118	A	O86a	+	-	-	-	-	-	-	-	-	-	-	+	-	+	-	-
E119	A	NA	+	-	-	-	-	-	-	-	-	-	-	-	-	+	-	-
E122	D	O167	+	-	-	-	-	-	-	-	-	-	-	+	-	-	-	-
E123	D	O1	+	-	-	-	-	-	-	-	-	-	+	+	-	+	-	+
E124	D	O146	-	-	-	-	-	-	-	-	-	-	-	-	-	-	-	-
Strain Totals (n=44)			34 77.3%	7 15.9%	3 6.8%	0 0%	0 0%	6 13.6%	5 11.4%	6 13.6%	4 9.1%	5 11.4%	16 36.4%	24 54.5%	0 0%	34 77.3%	4 9.1%	18 41%

Supplementary Table S4. The Egyptian *E. coli* draft genomes analysed in this study.

	Country of Isolation	Year of Isolation	Strain Name	Accession/ Reference	ST [c]	NDM [d]	OXA [d]	Notes	Ref
1	Egypt	ND [a]	51	DRR300282	ST1011			Human	[49]
2	Egypt	ND [a]	73	DRR300284	ST744			Human	[49]
3	Egypt	ND [a]	GCID_CRE_0002	SRR8291865	ST6355	NDM-1		Human: urine	[49]
4	Egypt	ND [a]	GCID_CRE_0004	SRR8291816	ST6355	NDM-1		Human: urine	[49]
5	Egypt	ND [a]	GCID_CRE_0007	SRR8291864	ST167	NDM		Human: urine	[49, 116]
6	Egypt	ND [a]	GCID_CRE_0011	SRR8291868	ST38			Human: urine	[49]
7	Egypt	ND [a]	GCID_CRE_0030	SRR8291879	ST216			Human: urine	[49]
8	Egypt	ND [a]	GCID_CRE_0033	SRR8291882	ST131			Human: urine	[49]
9	Egypt	ND [a]	GCID_CRE_0035	SRR8291883	ST405			Human: urine	[49]
10	Egypt	ND [a]	GCID_CRE_0036	SRR8291884	ST405			Human: urine	[49]
11	Egypt	ND [a]	GCID_CRE_0037	SRR8291889	ST450			Human: urine	[49]
12	Egypt	ND [a]	GCID_CRE_0038	SRR8291891	ST405			Human: urine	[49]
13	Egypt	ND [a]	GCID_CRE_0041	SRR8291887	ST216			Human: urine	[49]
14	Egypt	ND [a]	GCID_CRE_0043	SRR8291886	ST410			Human urine	[49]
15	Egypt	ND [a]	GCID_CRE_0044	SRR8291885	ST155			Human: urine	[49]
16	Egypt	2015	73G4	MKGV00000000	ST3541		OXA-244	Human	[117]
17	Egypt	2015	85H4	MKGW00000000	ST3541		OXA-244	Human: rectal swab	[117]
18	Egypt	2015	86J1	MKGU01000000	ST361		OXA-244	Human: rectal swab	[117]
19	Egypt	2016	345-HR128-ecoli_S20_L001	EB Assembly ESC_JB5542AA_AS	ST648			Human	[49]
20	Egypt	2016	356-HR3-ecoli_S19_L001	EB Assembly ESC_JB5541AA_AS	ST648			Dog	[49]
21	Egypt	2016	357-HR9-Ecoli_S16_L001	EB Assembly ESC_JB5543AA_AS	ST4553			Dog	[49]
22	Egypt	2016	358-HR-19-ecoli_S20_L001	EB Assembly ESC_JB5544AA_AS	ST997			Cat	[49]
23	Egypt	2016	359-HR20-ecoli_S20_L001	EB Assembly ESC_JB5545AA_AS	ST457			Dog	[49]
24	Egypt	2016	360-HR-26-ecoli_S21_L001	EB Assembly ESC_JB5548AA_AS	ST48			Cat	[49]
25	Egypt	2016	361-HR32-ecoli_S18_L001	EB Assembly ESC_JB5546AA_AS	ST167			Cat	[49]
26	Egypt	2016	362-HR108-Ecoli_S17_L001	EB Assembly ESC_JB5550AA_AS	ST48			Dog	[49]
27	Egypt	2016	363-HR-113-ecoli_S18_L001	EB Assembly ESC_JB5547AA_AS	ST38			Cat	[49]
28	Egypt	2016	364-HR115-ecoli_S2_L001	EB Assembly ESC_JB5549AA_AS	ST131			Cat	[49]
29	Egypt	2016	366-HR-132-ecoli_S19_L001	EB Assembly ESC_JB5552AA_AS	ST617			Human	[49]
30	Egypt	2016	367-HR-148-ecoli_S20_L001	EB Assembly ESC_JB5551AA_AS	ST167			Human	[49]
31	Egypt	2016	CFSAN061761	SRR5688551	ST10			Raw milk cow cheese	[49]
32	Egypt	2016	CFSAN061763	SRR5688549	ST38			Raw milk cow cheese	[49]
33	Egypt	2016	CFSAN061768	SRR5688545	ST38			Raw milk cow cheese	[49]
34	Egypt	2016	CFSAN061762	SRR5688550	ST10			Raw milk cow cheese	[49]
35	Egypt	2016	CFSAN061772	SRR5688556	ST10			Raw milk cow cheese	[49]
36	Egypt	2016	CFSAN061771	SRR5688554	ST1485			Raw milk cow cheese	[49]
37	Egypt	2016	CFSAN061766	CP042871 (chromosome only)	ST11075			Raw milk cow cheese	[49]
38	Egypt	2016	CFSAN061767	SRR5688546	ST361			Raw milk cow cheese	[49]
39	Egypt	2016	CFSAN061765	SRR5688547	ST1722		Oxa-244	Raw milk cow cheese	[49]
40	Egypt	2016	CFSAN061764	SRR5688548	ST1421			Raw milk cow cheese	[49]
41	Egypt	2016	CFSAN061760	SRR5688552	ST515			Raw milk cow cheese	[49]
42	Egypt	2016	CFSAN061759	SRR5688553	ST10			Raw milk cow cheese	[49]
43	Egypt	2016	CFSAN061770	SRR5688555	ST69			Raw milk cow cheese	[49]
44	Egypt	2015	SM_2209	JAJKRT010000000	ST167	NDM-5		Human: urine	[118]

45	Egypt	2016	SM_2947	JAJKPQ010000000	ST167	NDM-5	OXA-48	Human: urine	[118]
46	Egypt	2016	SM_2985	JAJKTY000000000	ST167	NDM-5		Human: abscess/ pus	[118]
47	Egypt	2016	SM_2763	JAJKQC010000000	ST410	NDM-5		Human: urine	[118]
48	Egypt	2016	SM_2761	JAJKQD010000000	ST410	NDM-5		Human: urine	[118]
49	Egypt	2016	SM_2766	JAJKUB010000000	ST410	NDM-5		Human: urine	[118]
50	Egypt	2016	SM_2771	JAJKQA010000000	ST410		Oxa-181	Human: urine	[118]
51	Egypt	2016	SM_2987	JAJKPK010000000	ST410	NDM-5		Human: abscess pus	[118]
52	Egypt	2016	SM_2815	JAJKPV010000000	ST410	NDM-5		Human: stomach tissue	[118]
53	Egypt	2016	SM_2799	JAJKPW010000000	ST410	NDM-5		Human: sputum	[118]
54	Egypt	2016	SM_2768	JAJKQB010000000	ST410	NDM-5		Human: urine	[118]
55	Egypt	2016	SM_2953	JAJKTZ010000000	ST410	NDM-5	Oxa-181	Human: urine	[118]
56	Egypt	2016	SM_2954	JAJKPO010000000	ST410	NDM-5	Oxa-181	Human: urine	[118]
57	Egypt	2016	SM_2955	JAJKPN010000000	ST410	NDM-5	Oxa-181	Human: urine	[118]
58	Egypt	2017	403-HR-3-ST_S9_L001	EB assembly ESC_JB5553AA_AS	ST43			Human	[49]
59	Egypt	2017	404-HR-20-ST_S10_L001	EB assembly ESC_JB5554AA_AS	ST10			Human	[49]
60	Egypt	2017	405-HR-23-ST_S11_L001	EB assembly ESC_JB5555AA_AS	ST226			Human	[49]
61	Egypt	2017	406-Hazem-4M_S24_L001	EB assembly ESC_JB5557AA_AS	ST226			Food	[49]
62	Egypt	2017	407-HR-17-M_S12_L001	EB assembly ESC_JB5556AA_AS	ST224			Food	[49]
63	Egypt	2017	408-HR-19-M_S13_L001	EB assembly ESC_JB5558AA_AS	ST1011			Food	[49]
64	Egypt	2017	409-HR-21-M_S14_L001	EB assembly ESC_JB5559AA_AS	ST48			Food	[49]
65	Egypt	2017	410-HR-3-CH_S15_L001	EB assembly ESC_JB5560AA_AS	ST10825			Food	[49]
66	Egypt	2017	411-HR-6CH_S15_L001	EB assembly ESC_JB5565AA_AS	ST48			Food	[49]
67	Egypt	2017	412-HR-71-CH_S16_L001	EB assembly ESC_JB5566AA_AS	ST156			Food	[49]
68	Egypt	2017	413-HR-82-CH_S17_L001	EB assembly ESC_JB5568AA_AS	ST224			Food	[49]
69	Egypt	2017	414-HR-2-M_S18_L001	EB assembly ESC_JB5567AA_AS	ST156			Food	[49]
70	Egypt	2017	415-HR-11-M_S19_L001	EB assembly ESC_JB5569AA_AS	ST58			Food	[49]
71	Egypt	2017	416-HR-4CH_S16_L001	EB assembly ESC_JB5570AA_AS	ST1011			Food	[49]
72	Egypt	2017	417-HR-59-CH_S20_L001	EB assembly ESC_JB5571AA_AS	ST155			Food	[49]
73	Egypt	2017	23ST	JACZFB010000000	ST226			Human: faeces:	[119]
74	Egypt	2017	E1_YT-HR	EB assembly ESC_UB8269AA_AS	ST2165			Milk	[49]
75	Egypt	2017	E2_YT-HR	EB assembly ESC_UB8285AA_AS	ST2165			Milk	[49]
76	Egypt	2017	EH-077	EB assembly ESC_UB8285AA_AS	ST224		Oxa-244	Milk	[49]
77	Egypt	2017	E1	SRR13933227	ST2165			Clinical mastitis: cow	[49]
78	Egypt	2017	E2	SRR13933226	ST2165			Subclinical mastitis: cow	[49]
79	Egypt	2018	E3	SRR13933221	ST2165			Raw cow milk	[49]
80	Egypt	2019	E4	SRR13933223	ST7624			Raw cow milk	[49]
81	Egypt	2019	M3-12-1	SRR23972359	ST1715	NDM-5		Red junglefowl: faeces	[49]
82	Egypt	2019	M3-15-1	SRR23972356	ST1715	NDM-5		Red junglefowl: faeces	[49]
83	Egypt	2019	M3-8-2	SRR23972354	ST1715	NDM-5		Red junglefowl: faeces	[49]
84	Egypt	2019	FDA1110442-S008-001	SRR9214239	ST8645			Sliced cheese	[49]
85	Egypt	2019	FDA1110442-S008-002	SRR9283224	ST8645			Sliced cheese	[49]
86	Egypt	2019	FDA1110442-S008-003	SRR9214240	ST8645			Sliced cheese	[49]
87	Egypt	2019	FDA1110442-S008-005	SRR9283108	ST8645			Sliced cheese	[49]
88	Egypt	2019	FDA1110442-S010-001	SRR9283105	ST216			Cheese	[49]
89	Egypt	2019	FDA00014286	EB assembly ESC_AB9029AA_AS	ST8645			Cheese	[49]
90	Egypt	2022	10	SRR35795447	ST410	NDM-5		Human	[49]
91	Egypt	2022	11	SRR35795446	ST167	NDM-5		Human	[49]
92	Egypt	2022	26	SRR35795445	ST131			Human	[49]

93	Egypt	2022	29	SRR35795444	ST167		Oxa-244	Human	[49]
94	Egypt	2022	21	SRR35795442	ST131			Human	[49]
95	Egypt	2023	Gene 25	JBIQPD010000000	ST167	NDM-5		Human	[48]
96	Egypt: Alexandria	2019	EGY_EC14142	SRR17067735	ST131			Human: urine	[54]
97	Egypt: Alexandria	2019	EGY_EC_13655	SRR19440483	ST410	NDM-5		Human: urine	[49]
98	Egypt: Alexandria	2020	98209	CP173532 (chromosome only)<br>CP173530.1 (plasmid)	ST410	NDM-5		Human	[48]
99	Egypt: Alexandria	2023	EC1	SRR33666352	ST648	NDM-5		Human: wound/ pus	[49]
100	Egypt: Alexandria	2023	EC2	SRR33666351	ST167	NDM-5		Human: wound/ pus	[49]
101	Egypt: Alexandria	2023	EC3	SRR33666360	ST1702	NDM-5		Human: wound/ pus	[49]
102	Egypt: Alexandria	2024	EC4	SRR33679778	ST167	NDM-5		Human: urine	[49]
103	Egypt: Alexandria	2023	EC5	SRR33679777	ST167	NDM-5		Human: urine	[49]
104	Egypt: Alexandria	2024	EC6	SRR33679774	ST167	NDM-5		Human: wound/ pus	[49]
105	Egypt: Alexandria	2023	EC7	SRR33679773	ST167	NDM-5		Human: blood	[49]
106	Egypt: Alexandria	2023	EC8	SRR33679772	ST167	NDM		Human: wound/ pus	[49]
107	Egypt: Alexandria	2023	EC9	SRR33679771	ST410	NDM-5		Human: urine	[49]
108	Egypt: Alexandria	2023	EC10	SRR33679770	ST167	NDM-5		Human: respiratory tract	[49]
109	Egypt: Alexandria	2023	EC11	SRR33679779	ST167	NDM-5		Human: urine	[49]
110	Egypt: Alexandria	2023	EC12	SRR33679769	ST167	NDM-5		Human: wound/pus	[49]
111	Egypt: Alexandria	2023	EC13	SRR33679768	ST167	NDM		Human: sputum	[49]
112	Egypt: Alexandria	2023	EC14	SRR33679776	ST167	NDM-5		Human: urine	[49]
113	Egypt: Alexandria	2023	EC15	SRR33679775	ST167	NDM-5		Human: urine	[49]
114	Egypt: Alexandria	2023	EC16	SRR33690897	ST--	NDM	OXA	Human: urine	[49]
115	Egypt: Alexandria	2023	EC17	SRR33690896	ST167			Human: blood	[49]
116	Egypt: Alexandria	2023	EC18	SRR33690892	ST167	NDM-5		Human: blood	[49]
117	Egypt: Alexandria	2024	EC19	SRR33690891	ST167	NDM-5		Human: respiratory tract	[49]
118	Egypt: Alexandria	2023	EC20	SRR33690890	ST15578	NDM-5		Human: blood	[49]
119	Egypt: Alexandria	2023	EC21	SRR33690889	ST167	NDM-5		Human: wound/ pus	[49]
120	Egypt: Alexandria	2023	EC22	SRR33690888	ST167	NDM-5		Human: blood	[49]
121	Egypt: Alexandria	2024	EC23	SRR33690887	ST167	NDM-5		Human: respiratory tract	[49]
122	Egypt: Alexandria	2024	EC24	SRR33690886	ST167	NDM-5		Human: respiratory tract	[49]
123	Egypt: Alexandria	2024	EC25	SRR33690885	ST167	NDM-5		Human: wound/pus	[49]
124	Egypt: Alexandria	2024	EC26	SRR33690895	ST167	NDM-5		Human: urine	[49]
125	Egypt: Alexandria	2024	EC27	SRR33690894	ST167	NDM-5		Human: wound/ pus	[49]
126	Egypt: Alexandria	2024	EC28	SRR33690893	ST167	NDM		Human: wound/ pus	[49]
127	Egypt: Assiut	2016	E4	SRR34956588	ST46		Oxa-244	Human: faeces	[64]
128	Egypt: Assiut	2016	E15	SRR34956587	ST167	NDM-5		Human: faeces	[64]
129	Egypt: Assiut	2016	E23	SRR34956586	ST167	NDM-19		Human: faeces	[64]
130	Egypt Assiut	2016	E27	JBQGWY000000000	ST410		OXA-181	Human: faeces	[64]
131	Egypt Assiut	2016	E28	JBQGWX010000000	ST617			Human: faeces	[64]
132	Egypt Assiut	2016	E29	SRR34956583	ST361			Human: faeces	[64]
133	Egypt Assiut	2016	E30	JBQGWV000000000	ST410			Human: faeces	[64]
134	Egypt Assiut	2016	E34	JBQGWU000000000	ST410			Human: faeces	[64]
135	Egypt: Assiut	2016	E35	SRR34956580	ST167	NDM-5		Human: faeces	[64]
136	Egypt: Assiut	2016	E36	JACEFX000000000	ST38			Human: faeces	[33]
137	Egypt: Assiut	2016	E42	JACEFW000000000	ST1380			Human: faeces	[33]
138	Egypt: Assiut	2016	E43	JBQGWS000000000	ST167	NDM-1		Human: faeces	[64]
139	Egypt: Assiut	2020	**E43**	JBXYYI000000000	ST131			Human: endotracheal aspirates	TW [b]

140	Egypt: Assiut	2020	**E106**	JBXYYH000000000	ST405	NDM-1		Human: endotracheal aspirates	TW [b]
141	Egypt: Assiut	2020	**E110**	JBXYYG000000000	ST131			Human: endotracheal aspirates	TW [b]
142	Egypt: Cairo	2022	A01	SRR31972389	ST405		OXA-181	Human: blood	[49, 53]
143	Egypt: Cairo	2022	A02	SRR31972388	ST501		OXA-244	Human: blood	[49, 53]
144	Egypt: Cairo	2022	A03	SRR31972377	ST1139		OXA-244	Human: blood	[49, 53]
145	Egypt: Cairo	2022	A04	SRR31972376	ST410		OXA-244	Human: blood	[49, 53]
146	Egypt: Cairo	2022	A05	SRR31972375	ST69		OXA-244	Human: blood	[49, 53]
147	Egypt: Cairo	2022	A06	SRR31972374	ST405		OXA-181	Human: blood	[49, 53]
148	Egypt: Cairo	2022	A07	SRR31972373	ST10		OXA-244	Human: blood	[49, 53]
149	Egypt: Cairo	2022	A08	SRR31972372	ST10		OXA-484	Human: blood	[49, 53]
150	Egypt: Cairo	2022	A09	SRR31972371	ST405		OXA-244	Human: blood	[49, 53]
151	Egypt: Cairo	2022	A10	SRR31972370	ST361		OXA-244	Human: blood	[49, 53]
152	Egypt: Cairo	2022	A11	SRR31972387	ST--		OXA-244	Human: blood	[49, 53]
153	Egypt: Cairo	2022	A12	SRR31972386	ST361		OXA-244	Human: blood	[49, 53]
154	Egypt: Cairo	2022	A13	SRR31972385	ST131		OXA-244	Human: blood	[49, 53]
155	Egypt: Cairo	2022	A14	SRR31972384	ST131		OXA-244	Human: blood	[49, 53]
156	Egypt: Cairo	2022	A15	SRR31972383	ST405		OXA-181	Human: blood	[49, 53]
157	Egypt: Cairo	2022	A16	SRR31972382	ST4981		OXA-244	Human: blood	[49, 53]
158	Egypt: Cairo	2022	A17	SRR31972381	ST155		OXA-181	Human: blood	[49, 53]
159	Egypt: Cairo	2022	A18	SRR31972380	ST167		OXA-244	Human: blood	[49, 53]
160	Egypt: Cairo	2022	A19	SRR31972379	ST3268		OXA-244	Human: blood	[49, 53]
161	Egypt: Cairo	2022	A20	SRR31972378	ST131		OXA-244	Human: blood	[49, 53]
162	Egypt Giza	2015	E2	CP048915.1 (chromosome only)	ST410			Human: ascitic fluid	[120]
163	Egypt: Mansoura	2016	HR119	QXNX00000000	ST167	NDM-5		Human	[121]
164	Egypt: Mansoura	2016	HR135	QXNW00000000	ST361	NDM-1		Human	[121]
165	Egypt: Mansoura	2016	HR163	QXNV00000000	ST410	NDM-5	Oxa-181	Dog Environment	[121]
166	Egypt: Mansoura	2016	HR164	QXNU00000000	ST410	NDM-5	Oxa-181	Dog Environment	[121]
167	Egypt: Mansoura	2016	HR165	QXNT00000000	ST410	NDM-5	Oxa-181	Dog Environment	[121]
168	Egypt: Mansoura	2016	HR166	QXNS01000000	ST410	NDM-5	Oxa-181	Dog Environment	[121]
169	Egypt: Mansoura	2016	HR167	QXNR01000000	ST410	NDM-5	Oxa-181	Dog Environment	[121]
170	Egypt: Mansoura	2016	HR169	QXNQ00000000	ST410	NDM-5	Oxa-181	Dog Environment	[121]
171	Egypt: Mansoura	2016	HR170	QXNP01000001	ST410	NDM-5	Oxa-181	Dog Environment	[121]
172	Egypt: Sidi Ghazy	2019	A-1-4-1	SRR13675950	ST155			Chicken: faeces	[49, 122]
173	Egypt: Sidi Ghazy	2019	A-1-8-1	SRR13675949	ST155			Chicken: faeces	[49, 122]
174	Egypt: Sidi Ghazy	2019	A-1-10-1	SRR13675951	ST--			Chicken: faeces	[49, 122]
175	Egypt: Sidi Ghazy	2019	A-1-11-3	SRR13675948	ST--			Chicken: faeces	[49, 122]
176	Egypt: Sidi Ghazy	2019	A-1-22-2	SRR13675947	ST--			Chicken: faeces	[49, 122]
177	Egypt: Sidi Ghazy	2019	M2-13-1	SRR23972352	ST167	NDM-19		Chicken: faeces	[123]
178	Egypt Tanta	ND [a]	71	SRR8885623	ST410			Human	[48]

[a] ND, not determined. [b] TW, this work.

Software at the Center for Genomic Epidemiology (CGE) (http://www.genomicepidemiology.org/ (accessed on 25 March 2026)) and Solu Genomics (https://www.solugenomics.com/ (accessed on 25 March 2026)) was used to identify: [c] the sequence type (ST) [37, 50] and [d] the presence of carbapenemase genes [50, 40].

Supplementary Table S5. The *E. coli* ST131 genomes used for phylogenetic SNP analysis in this study.

	Country of Isolation	Year of Isolation	Strain Name	Accession/ Reference	ST[d]	OXA [e]	KPC [e]	Notes	Ref
1	UK	2007	S2EC	ERR161235	ST131-A [b]			Human: urine	[65]
2	UK	2009	S26EC	ERR161245	ST131-A [b]			Human: urine	[65]
3	UK	2009	S37EC	ERR161302	ST131-A [b]			Human: urine	[65]
4	New Zealand	2009	S94EC	ERR161257	ST131-A [b]			Human: blood	[65]
5	Canada	2009	S120EC	ERR161283	ST131-A [b]			Human: blood	[65]
6	Japan	2012	KK85	DRR092880	ST131-A [b]			Human	[66]
7	UK	2009	S19EC	ERR161241	ST131-B [b]			Human: urine	[65]
8	Australia	2009	S79EC	ERR161305	ST131-B [b]			Human: urine	[65]
9	Spain	2010	HVM1147	ERR161318	ST131-B [b]			Human: abscess	[65]
10	Spain	2010	HVM2044	ERR161323	ST131-B [b]			Human: blood	[65]
11	Australia	2011	S114EC	ERR161277	ST131-B [b]			Human: urine	[65]
12	Germany	2012	AZ844071	SRR2970655	ST131-B [b]			Human: respiratory	[66]
13	Unknown	ND [a]	VRES0207	ERR998794	ST131-C1 [b]			Unknown	[66]
14	Unknown	ND [a]	VRES0204	ERR998792	ST131-C1 [b]			Unknown	[66]
15	Spain	2010	HVR83	ERR161311	ST131-C1 [b]			Human: blood	[65]
16	Spain	2011	P56EC	ERR161310	ST131-C1 [b]			Human: rectal swab	[65]
17	UK	2011	S117EC	ERR161280	ST131-C1 [b]			Human: sepsis	[65]
18	USA	2013	BIDMC 63	SRR1180699	ST131-C1 [b]			Human: swab	[66]
19	Australia	2015	MER-94	SRR5936488	ST131-C2 [b]			Human: blood	[66]
20	Germany	2016	144	ERR2232739	ST131-C2 [b]			Human	[66]
21	Ireland	2016	ME160526	ERR1981378	ST131-C2 [b]	OXA-48		Human	[66]
22	Sweden	2018	R13	SRR22405941	ST131-C2 [b]				[62]
23	Sweden	2018	S9	SRR22405918	ST131-C2 [b]				[62]
24	Unknown	ND [a]	MDRAPT12985849	ERR12184154	ST131			Unknown	[48]
25	Unknown	ND [a]	MDRAPT13217741	ERR12419656	ST131			Unknown	[48]
26	Netherlands	ND [a]	SCK63-03	ERR1617958	ST131			Human	[48]
27	UK	2014	AMC_113	SRR10271587	ST131			Human	[48, 124]
28	Sweden	2015	EF538	ERR4426100	ST131			Human	[48]
29	USA	2016	1449	CP184069.1 (Chromosome)<br>CP184072.1 (p1449_3)	ST131		KPC-2	Human	[48]
30	France	2018	BLSE2018-69	ERR5037342	ST131			Human	[48, 124]
31	Switzerland	2018	721474-18	ERR10177563	ST131			Human: urine	[48, 124]
32	Norway	2019	3b968aa8-0c40-11ee-a825-fa163eea3084	ERR13369462	ST131			Unknown	[48]
33	Japan	2020	JBAGABF-19-0051	DRR386351	ST131			Human: blood	[124]
34	Latvia	2021	L12	SRR19268185	ST131			Human: blood	[48]
35	Denmark	2021	AMA004015	ERR12945254	ST131	OXA-244		Human	[124]
36	USA	2021	2021JQ-00479	SRR18680400	ST131	OXA-244		Human: blood	[48]
37	Netherlands	2023	RIVM_C055770	SRR27977116	ST131	OXA-244		Human: urine	[124]
38	Egypt	ND [a]	GCID_CRE_0033	SRR8291882	ST131			Human: urine	[49]
39	Egypt	2016	364-HR115-ecoli_S2_L001	EB Assembly ESC_JB5549AA_AS	ST131			Cat	[49]
40	Egypt	2022	26	SRR35795445	ST131			Human	[49]

41	Egypt	2022	21	SRR35795442	ST131			Human	[49]
42	Egypt: Alexandria	2019	EGY_EC14142	SRR17067735	ST131			Human: urine	[54]
43	Egypt: Assiut	2020	**E43**	JBXYYI000000000	ST131			Human: endotracheal aspirates	TW [c]
44	Egypt: Assiut	2020	**E110**	JBXYYG000000000	ST131			Human: endotracheal aspirates	TW [c]
45	Egypt: Cairo	2022	A13	SRR31972385	ST131	OXA-244		Human: blood	[49, 53]
46	Egypt: Cairo	2022	A14	SRR31972384	ST131	OXA-244		Human: blood	[49, 53]
47	Egypt: Cairo	2022	A20	SRR31972378	ST131	OXA-244		Human: blood	[49, 53]

[a] ND, not determined. [b] ST131 reference strain. [c] TW, this work.

Software at the Center for Genomic Epidemiology (CGE) (http://www.genomicepidemiology.org/ (accessed on 25 March 2026)) and Solu Genomics (https://www.solugenomics.com/ (accessed on 25 March 2026)) was used to identify: [d] the sequence type (ST) [37, 50] and [e] the presence of carbapenemase genes [50, 40].

Supplementary Table S6. The *E. coli* ST405 genomes used for phylogenetic SNP analysis in this study.

	Country of Isolation	Year of Isolation	Strain Name	Accession/ Reference	ST [d]	NDM [e]	OXA [e]	Notes	Ref
1	Canada	2014	FDAARGOS_448	SRR5884862	ST405-Ref[b]	NDM-5			[70]
2	China	2015	WCHEC96200	SRR6474930	ST405-Ref[b]	NDM-4		rectal swab	[52, 125]
3	China	2017	WCHEC040047	SRR7026309	ST405-Ref[b]	NDM-5			[70]
4	Myanmar	2015	M101	DRR140909	ST405-Ref[b]	NDM-5			[124, 70]
5	Myanmar	2015	M105	DRR140910	ST405-Ref[b]	NDM-5			[70, 126]
6	Myanmar	2016	M422	DRR111582	ST405-Ref[b]	NDM-5			[124, 70]
7	Myanmar	2016	M506	DRR111588	ST405-Ref[b]	NDM-5			[124, 70]
8	Myanmar	2016	M516	DRR111596	ST405-Ref[b]	NDM-5			[124, 70]
9	Myanmar	2016	M622W	DRR140902	ST405-Ref[b]	NDM-5			[124, 70]
10	Myanmar	2016	M520B	DRR111600	ST405-Ref[b]	NDM-5			[124, 70]
11	Nepal	2013	IOMTU548	DRR075627	ST405-Ref[b]	NDM-5			[124, 70]
12	Nepal	2013	IOMTU605	DRR075634	ST405-Ref[b]	NDM-5			[124, 70]
13	UK	2018	467367	SRX4174605	ST405-Ref[b]	NDM-5			[70]
14	USA	2014	CRE17	SRR6793840	ST405-Ref[b]	NDM-5			[70]
15	USA	2015	222	SRR3615373	ST405-Ref[b]	NDM-5			[70]
16	USA	2016	CRE87	SRR6793823	ST405-Ref[b]				[70]
17	USA	2017	PNUSAE010746	SRR6297323	ST405-Ref[b]	NDM-5			[70]
18	USA	2018	PNUSAE021121	SRR8378671	ST405-Ref[b]	NDM-5			[70]
19	Egypt	ND [a]	GCID_CRE_0035	SRR8291883	ST405			Human: urine	[49]
20	Egypt	ND [a]	GCID_CRE_0036	SRR8291884	ST405			Human: urine	[49]
21	Egypt	ND [a]	GCID_CRE_0038	SRR8291891	ST405			Human: urine	[49]
22	Egypt: Assiut	2020	**E106**	JBXYYH000000000	ST405	NDM-1		Human: endotracheal aspirates	TW [c]
23	Egypt: Cairo	2022	A01	SRR31972389	ST405		OXA-181	Human: blood	[49, 53]
24	Egypt: Cairo	2022	A06	SRR31972374	ST405		OXA-181	Human: blood	[49, 53]
25	Egypt: Cairo	2022	A09	SRR31972371	ST405		OXA-244	Human: blood	[49, 53]
26	Egypt: Cairo	2022	A15	SRR31972383	ST405		OXA-181	Human: blood	[49, 53]
27	Ethiopia	2016	BW-198	SRR22225545	ST405				[48, 124]
28	Australia	2017	AUSMDU00010797	SRR14673687	ST405				[48, 124]
29	Ethiopia	2019	186	SRR17179348	ST405				[48, 124]
30	Germany	ND [a]	17e	ERR2348786	ST405				NCBI
31	Malawi	ND [a]	ERS7957904	ERR10893708	ST405				[48, 124]
32	UK	ND [a]	146991	SRR18429231	ST405				[48, 124]
33	Unknown	ND [a]	64678	ERR1640728	ST405				[48, 124]

[a] ND, not determined. [b] ST405 reference strain. [c] TW, this work.

Software at the Center for Genomic Epidemiology (CGE) (http://www.genomicepidemiology.org/ (accessed on 25 March 2026)) and Solu Genomics (https://www.solugenomics.com/ (accessed on 25 March 2026)) was used to identify: [d] the sequence type (ST) [37, 50] and [e] the presence of carbapenemase genes [50, 40].

Supplementary Figure legends

Figure S1. PCR analysis of ESBL genes carried by *E. coli* isolates. **A)** The panel shows the gel electrophoresis of PCR-amplified products for *bla*$_{CTX\text{-}M\text{-}3}$-like genes. The agarose gel was loaded as follows: lane M, DNA marker; lanes 1 to 13, *bla*$_{CTX\text{-}M\text{-}3}$ positive isolates; lane 14, *bla*$_{CTX\text{-}M\text{-}3}$ positive control; lane 15, negative control. **B)** Gel electrophoresis of PCR-amplified products for *bla*$_{CTX\text{-}M\text{-}3}$-like genes used for DNA sequencing. The agarose gel was loaded as follows: lane M, DNA marker; lanes 1 to 6, *bla*$_{CTX\text{-}M\text{-}3}$ positive isolates; lane 7, *bla*$_{CTX\text{-}M\text{-}3}$ positive control; lane 8, negative control. **C)** Gel electrophoresis of PCR-amplified products for *bla*$_{CTX\text{-}M\text{-}14}$-like genes. The agarose gel was loaded as follows: lane M, DNA marker; lane 1, *bla*$_{CTX\text{-}M\text{-}14}$ positive isolate; lane 2, *bla*$_{CTX\text{-}M\text{-}14}$ positive control; lane 3, negative control. **D)** Gel electrophoresis of PCR-amplified products of *bla*$_{CTX\text{-}M\text{-}14}$ like genes used for DNA sequencing. The gel was loaded: lane M, DNA marker; lanes 1 to 5, *bla*$_{CTX\text{-}M\text{-}14}$ positive isolates; lane 6, *bla*$_{CTX\text{-}M\text{-}14}$ positive control; lane 7, negative control. **E)** DNA gel electrophoresis of the PCR-amplified products of *bla*$_{TEM}$. The agarose gel was loaded as follows: lane M, DNA marker; lanes 1 and 2, *bla*$_{TEM}$ positive isolates; lane 3, *bla*$_{TEM}$ positive control; lane 4, negative control. **F)** Gel electrophoresis of PCR-amplified products of *bla*$_{TEM}$ used for DNA sequencing. The gel was loaded: lane M, DNA marker; lanes 1 to 3 and 8, *bla*$_{TEM}$ negative isolates; Lane 4 to 7, *bla*$_{TEM}$ positive isolates; lane 9, positive control; lane 10, negative control. **G)** Gel electrophoresis of the PCR-amplified products of *bla*$_{SHV}$. The gel was loaded: lane M, DNA marker; lane 1, *bla*$_{SHV}$ positive control; lane 2, *bla*$_{SHV}$ positive isolate; lane 3, negative control. **H)** Gel electrophoresis of PCR-amplified products of *bla*$_{SHV}$ used for DNA sequencing. The gel was loaded as follows: lane M, DNA marker; lane 1, negative control; lane 2, *bla*$_{SHV}$ positive control; lane 3, *bla*$_{SHV}$ positive isolate.

Figure S2. PCR analysis of carbapenemase genes carried by *E. coli* isolates. **A)** The panel shows gel electrophoresis of PCR-amplified products for the *bla*$_{NDM\text{-}1}$ gene. The gel was loaded as follows: lane M, DNA marker; lanes 1 to 3, *bla*$_{NDM\text{-}1}$ positive isolates; lane 4, *bla*$_{NDM\text{-}1}$ positive control; lane 5, *bla*$_{NDM\text{-}1}$ negative control. **B)** Gel electrophoresis of the PCR-amplified products for the *bla*$_{KPC}$ gene. The gel was loaded: lanes 1 to 4 and 6 to 8, *bla*$_{KPC}$ negative isolates; lanes 5, 9 and10, *bla*$_{KPC}$ positive controls (839 bp); lanes 11 and 12, *bla*$_{KPC}$ negative controls; lane M, DNA marker. **C)** Gel electrophoresis of the PCR-amplified products for the *bla*$_{VIM}$ gene. The agarose gel was loaded as follows; lane 1 to 8, *bla*$_{VIM}$ negative isolates; lane 9, negative control; lane 10, *bla*$_{VIM}$ positive control; lane M, DNA marker.

Figure S3. PCR analysis of *E. coli* virulence genes carried by *E. coli* isolates. **A)** The panel shows gel electrophoresis of the PCR-amplified products for the *fimH* gene. The gel was loaded as follows: lane 1, DNA marker; lane 2, negative control; lane 3, *fimH* positive control; lanes 4 to 9, *fimH* positive isolates. **B)**

Gel electrophoresis of the PCR-amplified products for *fyuA*. The gel was loaded: lane 1, DNA marker; lanes 2 and 3, *fyuA* positive isolates; lane 4, *fyuA* negative isolate; lane 5, *fyuA* positive control; lane 6, negative control. **C)** Gel electrophoresis of the PCR-amplified products for the *hylA* gene. The agarose gel was loaded as follows; lane 1, DNA marker; lanes 2 to 4, *hylA* negative isolates; lane 5, *hylA* positive isolate; lane 6, negative control; lane 7, *hylA* positive control. **D)** Gel electrophoresis of the PCR-amplified products for *traT, iutA* and *papA* genes. The gel was loaded: lane 1, DNA marker; lanes 2 to 5, isolates carrying *traT* (280 bp); lane 4, isolate carrying *papA* (650 bp); lane 5, strain carrying *iutA* (360 bp). **E)** Gel electrophoresis of the PCR-amplified products for the *papGIII, kpsMTII* and *sfa/focDE* genes. The gel was loaded as follows: lane 1: DNA marker; lanes 2 to 4, *sfa/focDE* carrying strains (410 bp); lanes 2 and 3, *papGIII* carrying strains (225 bp); lanes 4 to 7, *kpsMTII* carrying control strains (272 bp).

Figure S4. Comparison of *E. coli* strain R13 plasmid pR13-1180 with the draft genome of *E. coli* E43. **A)** The panel shows the comparison of plasmid pR13-1180 (CP107152.1: human isolate) [62] with the draft genome of E43 and E43 contigs 21 (Col156: 38,235 bp), 26 (IncFIA: 25,370 bp), 27 (IncFII: 24,910 bp), 31 (IncFIB: 11,917 bp) and 36 (IncFII: 10,220 bp) using ProkSee [46]. The outer two rings display the genes of pR13-1180 (CDS) on both strands. The green, light green, brown, purple, blue and pink rings illustrate the BLAST results when the E43 draft genome and contigs 21, 26, 27, 31 and 36, respectively, are compared to pR13-1180. **B)** Analysis of contig 27 from *E. coli* strain E43. Genomic organisation of E43 contig 27 (24,910 bp) using ProkSee [46]. The location of ARG is indicated. **C)** The panel shows the comparison of plasmid pR13-1180 (CP107152.1) [62] with plasmid pS9-S4K58-1 (human isolate: CP107123.1) [62] and the draft genomes of *E. coli* strains E43, BLSE2018-69 (human isolate: ERR5037342), 721474-18 (human isolate: ERR10177563), SCK63-03 (human isolate: ERR1617958), L12 (human isolate: SRR19268185), AMC_113 (human isolate: SRR10271587), EF538 (human isolate: ERR4426100) and 3b968aa8-0c40-11ee-a825-fa163eea3084 (isolation source unknown: ERR13369462) using ProkSee [46]. The outer two rings display the genes of pR13-1180 (CDS) on both strands. The coloured bands illustrate the BLAST results when each DNA seqeunce is compared to pR13-1180. In both panels, the location of ARG carried by pR13-1180, as well as the IncFIA, IncFIB, two IncFII and Col156 replicons, is indicated.

Figure S5. Analysis of contigs 21 and 24 from *E. coli* strain E106. Genomic organisation of E106. **A)** contig 21 (79,866 bp) and **B)** contig 24 (65,526 bp) using ProkSee [46]. The location of ARG and specific plasmid replicons, is indicated. **C)** Comparison of plasmid pCVM29188_146 from *Salmonella enterica* servovar Kentucky CVM29188 (poultry: CP001122.1) [63] with E106 contigs 21 and 24 using ACT [47]. Alignment is shown by red and blue banding.

Figure S6. Comparison of *E. coli* strain 1449 plasmid p1449_1 with the draft genome of *E. coli* E110. **A)** The panel shows the comparison of plasmid p1449_1 (CP184070.1: human isolate) with the draft genome of E110 and E110 contigs 36 (IncFIA: 15,974 bp), 38 (IncFIB: 15,234 bp), 41 (Col156: 10,625 bp) and 47 (IncFII: 7,747 bp) using ProkSee [46]. The outer two rings display the genes of p1149_1 (CDS) on both strands. The green, light green, brown, purple and blue rings illustrate the BLAST results when the E110 draft genome and contigs 36, 38, 41 and 47, respectively, are compared to p1449_1.

Supplementary Figure S1.

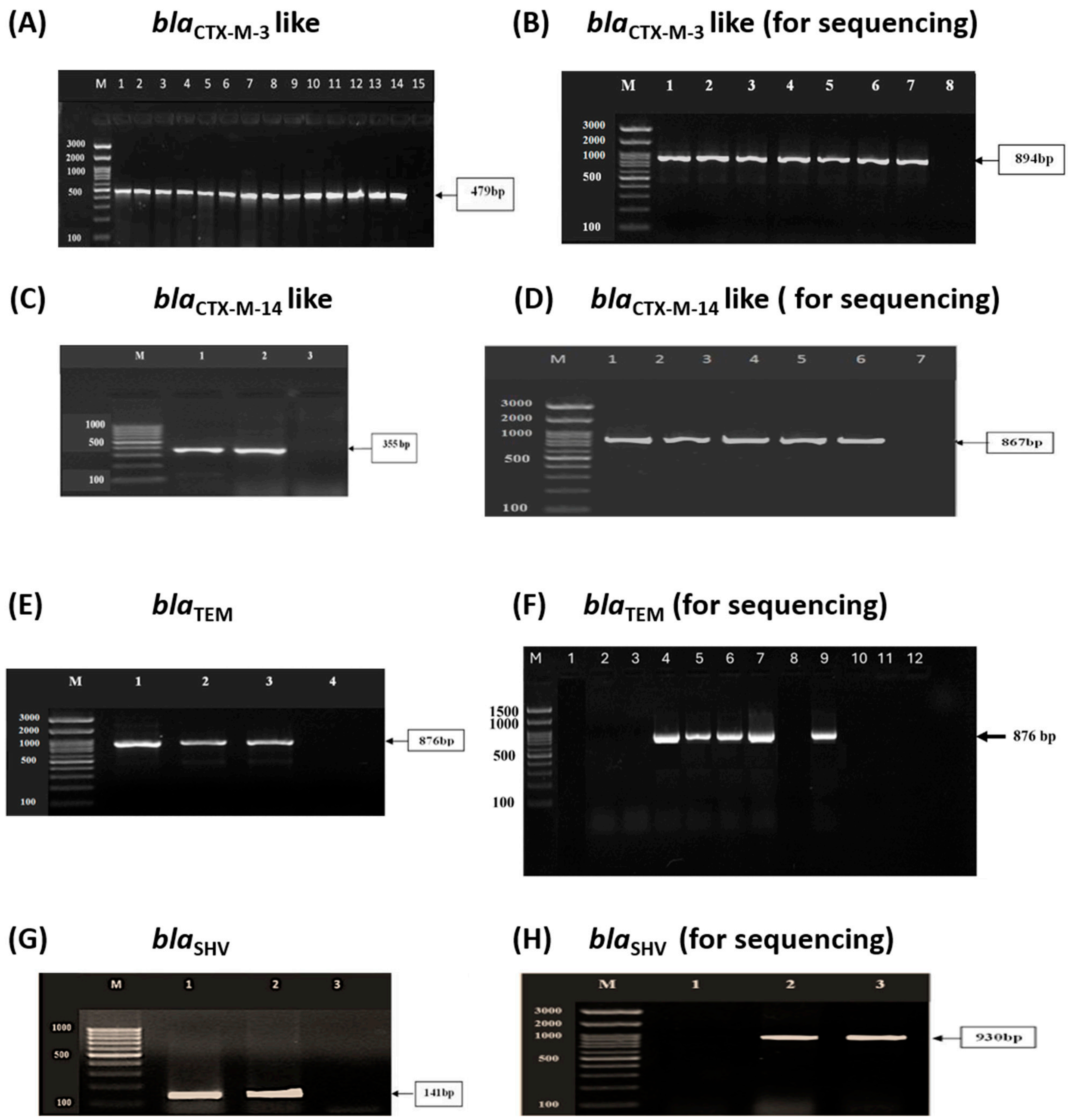

Supplementary Figure S2.

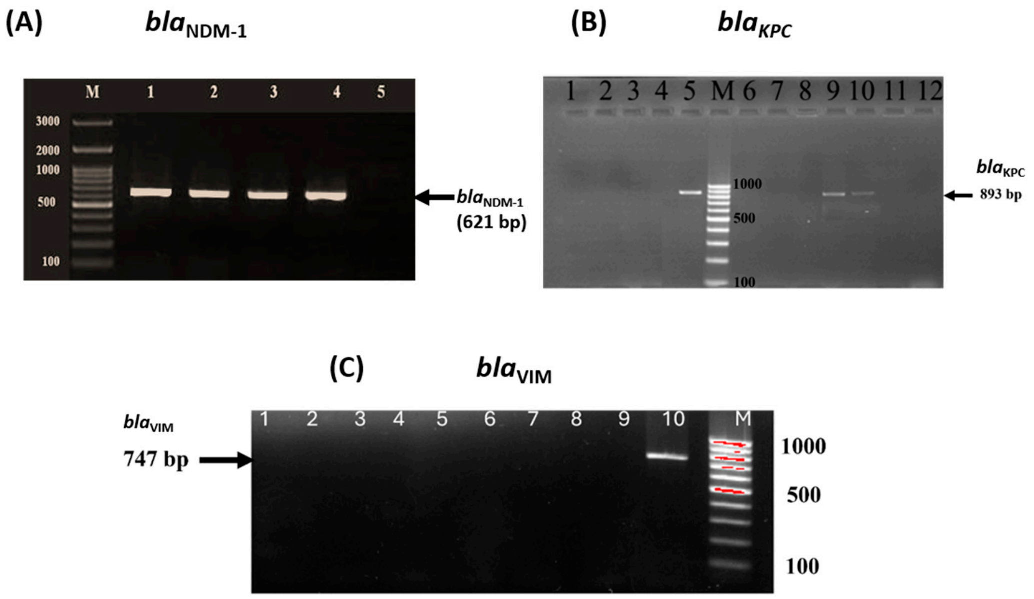

Supplementary Figure S3.

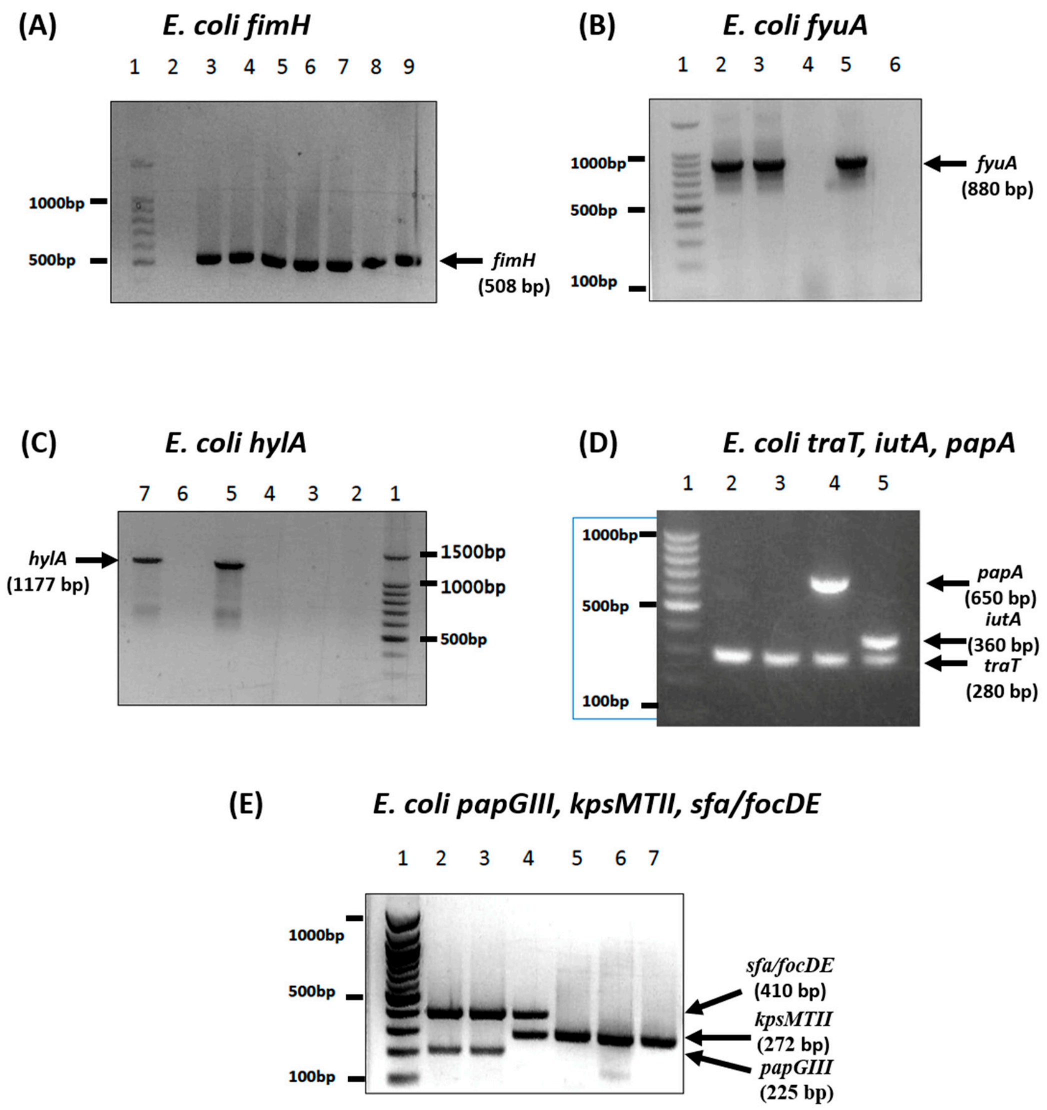

Supplementary Figure S4.

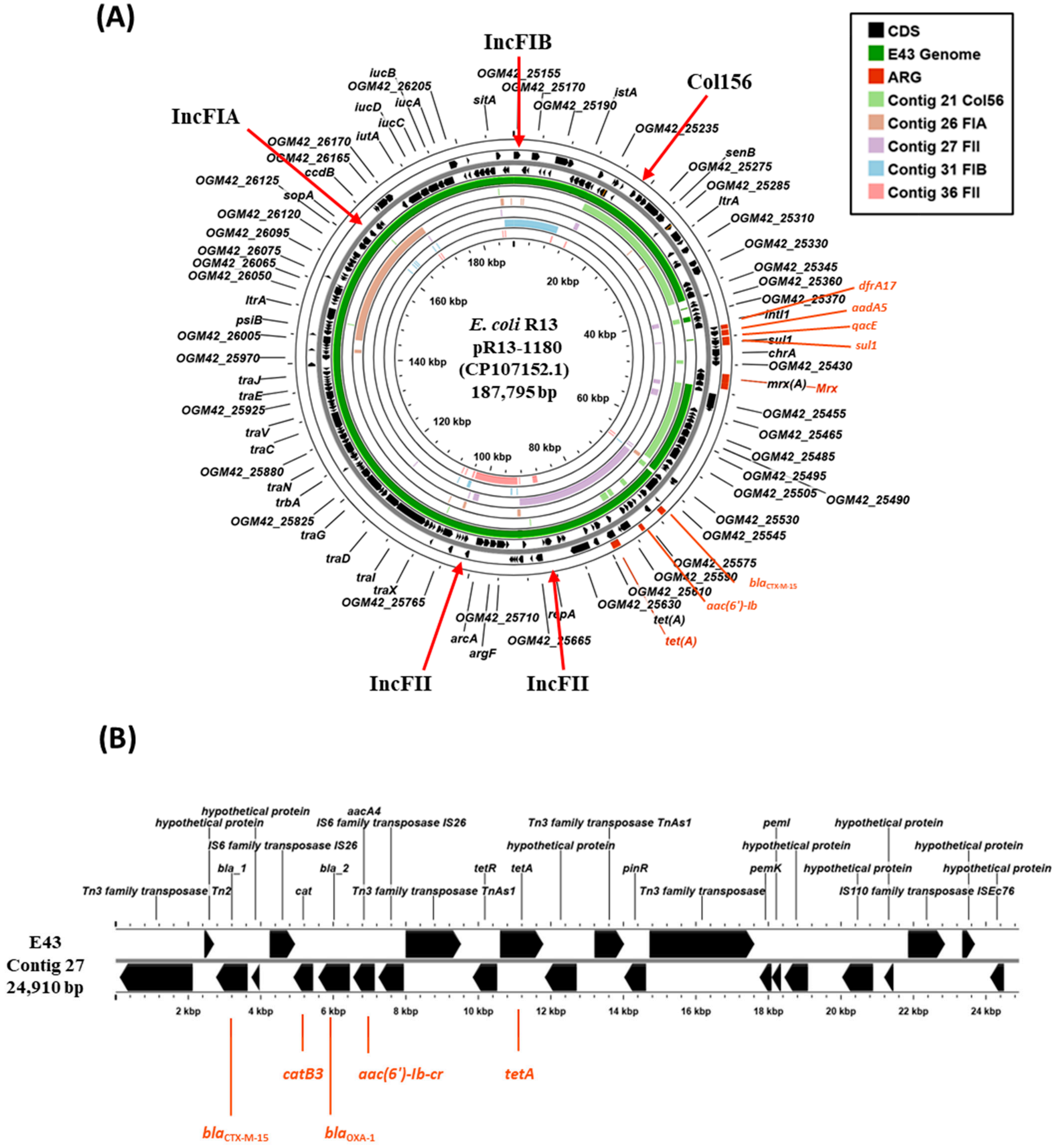

Supplementary Figure S4 Continued.

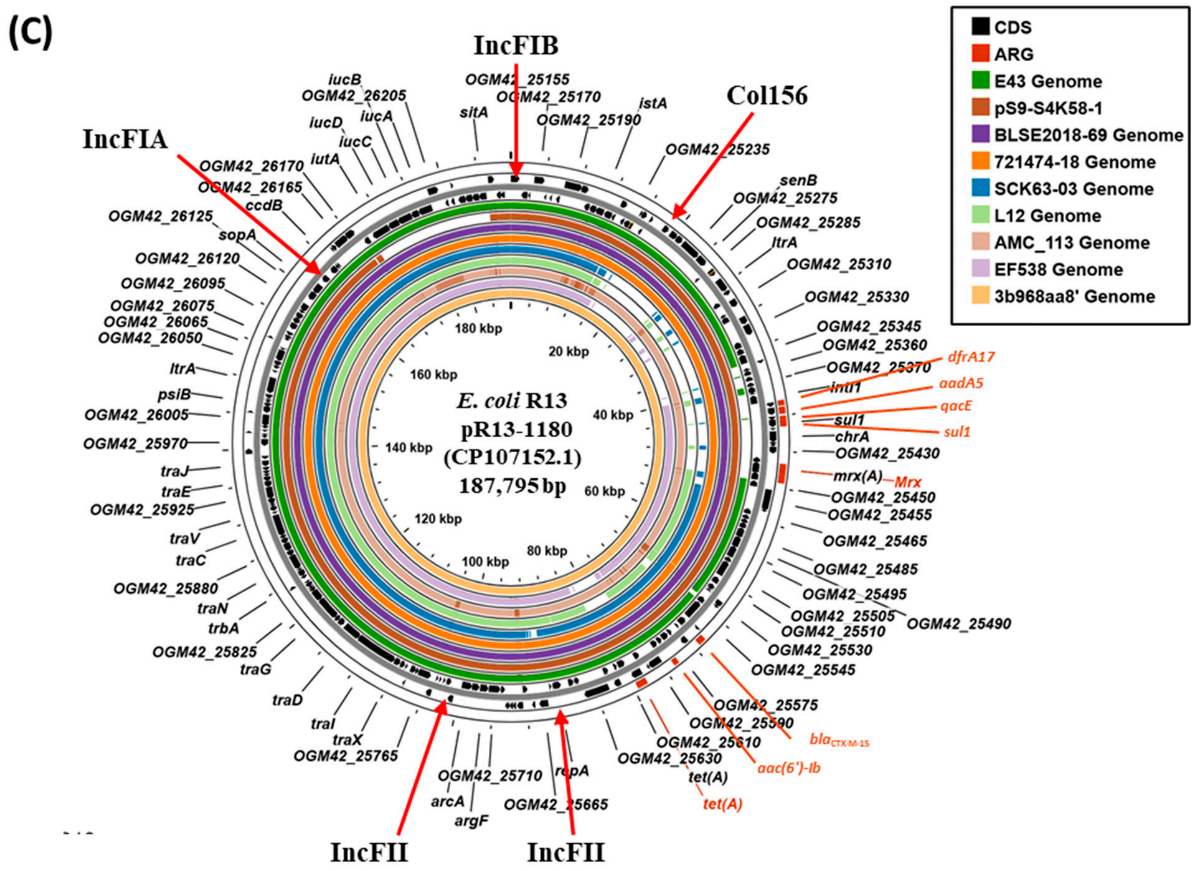

Supplementary Figure S5.

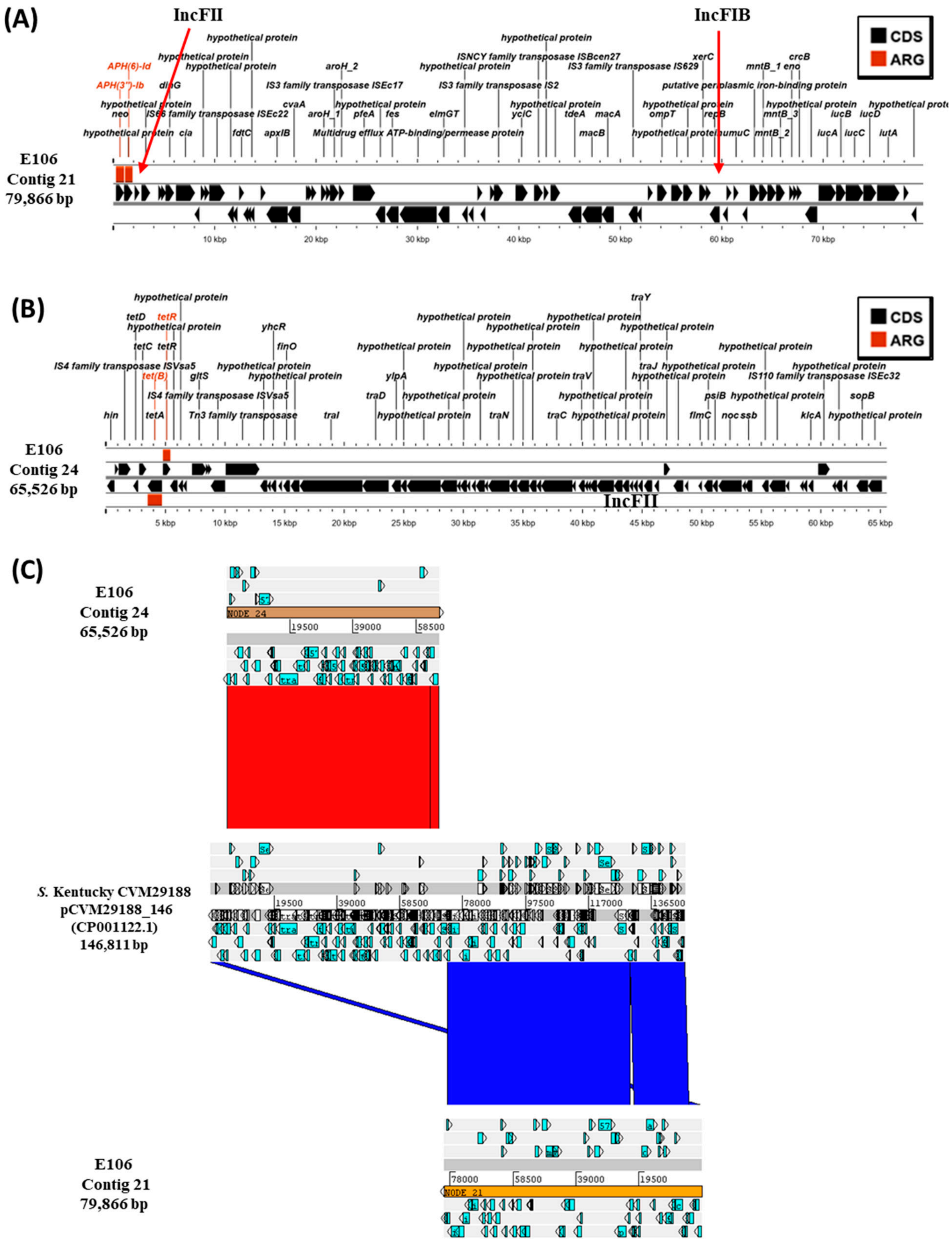

Supplementary Figure S6.

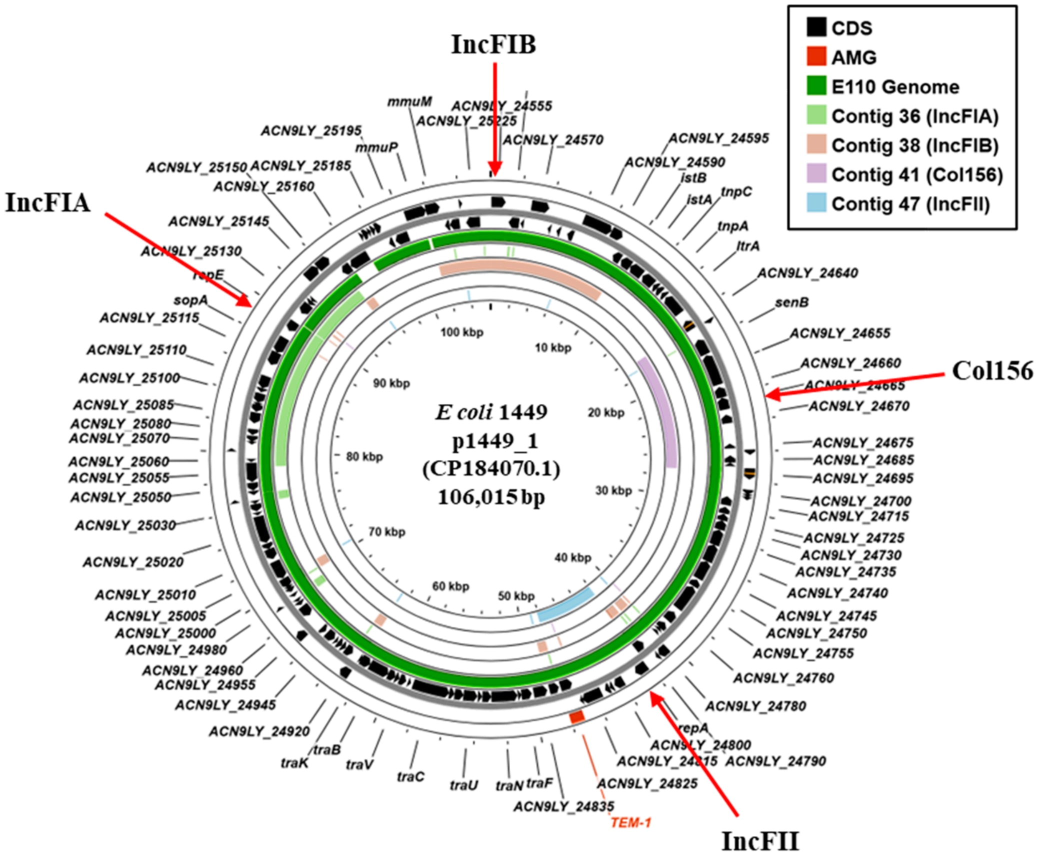

Supplement: Supplementary file 1 [file microorganisms-14-01438-s001.zip › microorganisms-4360277-supplementary.pdf]
